# Identification of multidrug chemoresistant genes in head and neck squamous cell carcinoma cells

**DOI:** 10.1186/s12943-023-01846-3

**Published:** 2023-09-04

**Authors:** Neha Khera, Asvika Soodhalaagunta Rajkumar, Khlood Abdulkader M Alkurdi, Zhiao Liu, Hong Ma, Ahmad Waseem, Muy-Teck Teh

**Affiliations:** 1 Centre for Oral Immunobiology and Regenerative Medicine, Institute of Dentistry, Barts and The London School of Medicine and Dentistry, Queen Mary University of London, The Blizard Building, 4, Newark Street, London, E1 2AT UK; 2 China-British Joint Molecular Head and Neck Cancer Research Laboratory, Affiliated Stomatological Hospital of Guizhou Medical University, Guizhou, China

**Keywords:** Multidrug chemoresistance, Chemosensitization, Cisplatin, Drug-gene interaction, INHBA, NEK2, Squamous cell carcinomas, Oral cancer

## Abstract

Multidrug resistance renders treatment failure in a large proportion of head and neck squamous cell carcinoma (HNSCC) patients that require multimodal therapy involving chemotherapy in conjunction with surgery and/or radiotherapy. Molecular events conferring chemoresistance remain unclear. Through transcriptome datamining, 28 genes were subjected to pharmacological and siRNA rescue functional assays on 12 strains of chemoresistant cell lines each against cisplatin, 5-fluorouracil (5FU), paclitaxel (PTX) and docetaxel (DTX). Ten multidrug chemoresistance genes (TOP2A, DNMT1, INHBA, CXCL8, NEK2, FOXO6, VIM, FOXM1B, NR3C1 and BIRC5) were identified. Of these, four genes (TOP2A, DNMT1, INHBA and NEK2) were upregulated in an HNSCC patient cohort (n = 221). Silencing NEK2 abrogated chemoresistance in all drug-resistant cell strains. INHBA and TOP2A were found to confer chemoresistance in majority of the drug-resistant cell strains whereas DNMT1 showed heterogeneous results. Pan-cancer Kaplan-Meier survival analysis on 21 human cancer types revealed significant prognostic values for INHBA and NEK2 in at least 16 cancer types. Drug library screens identified two compounds (Sirodesmin A and Carfilzomib) targeting both INHBA and NEK2 and re-sensitised cisplatin-resistant cells. We have provided the first evidence for NEK2 and INHBA in conferring chemoresistance in HNSCC cells and siRNA gene silencing of either gene abrogated multidrug chemoresistance. The two existing compounds could be repurposed to counteract cisplatin chemoresistance in HNSCC. This finding may lead to novel personalised biomarker-linked therapeutics that can prevent and/or abrogate chemoresistance in HNSCC and other tumour types with elevated NEK2 and INHBA expression. Further investigation is necessary to delineate their signalling mechanisms in tumour chemoresistance.

**Supplementary Information:**

The online version contains supplementary material available at 10.1186/s12943-023-01846-3.

## Background

Head and neck squamous cell carcinomas (HNSCCs) constitute 90% of all head and neck cancers. Whilst a minority of HNSCCs are caused by human papilloma virus (HPV) infection the majority of the more aggressive HPV-negative HNSCCs (75%) are associated with tobacco and alcohol use [1]. Although the cure rate of HNSCC patients with early-stage disease treated with primary surgery and/or radiotherapy has been excellent (70–90% 5-year overall survival) [1]; unfortunately, two-thirds of HNSCC patients present with advanced-stage disease suffer from poor survival outcome due to limited treatment options and/or treatment failure. Hence, the long-term survival rate of HNSCC patients remains unchanged over many decades at about 50% despite advancements in treatment modalities [1].

For HNSCC patients requiring multimodal therapy involving chemotherapy, cisplatin, 5-fluorouracil (5FU) and paclitaxel (PTX) or docetaxel (DTX) are amongst the most commonly used chemotherapeutic agents often used in combinations [1, 2]. Unfortunately, treatment failure due to development of resistance to chemo and/or radiotherapy remains a major cause of HNSCC poor survival rates. Unlike lung and breast cancer patients, all HNSCC patients are treated with almost the same combinations of treatment irrespective of the genetic makeup of their cancer. This is mainly due to poor understanding of molecular heterogeneity of HNSCC. Research into molecular biomarkers that can stratify sub-populations and indicate the most suitable intervention based on individual patient’s tumour molecular profile would reduce toxicity, improve morbidity and treatment outcome [1, 3].

A number of key mechanisms for conferring intrinsic chemoresistance in HNSCC tumour cells have been studied and these include perturbations of pathways regulating apoptosis/cell death, DNA damage repair, epithelial mesenchymal transition, cell cycle, cancer stem cell, chromatin/epigenetic, miRNA processing, autophagy and stroma/matrix, immune cell interactions [4]. Although a number of molecular markers have been proposed for counteracting chemoresistance in HNSCC [4], exploitation of molecular markers for risk stratification in HNSCC patients prior to treatment decision largely remains at infancy [1, 3].

This study explored using a combination of bioinformatics transcriptome data mining, differential gene expression analysis in chemoresistant cell line models and validation in clinical HNSCC tumour specimens, pharmacological dose-response drug library screen and cell culture models with the aim to identify key multidrug-resistant biomarker genes and repurpose existing drugs to counteract chemoresistance.

## Methods

All details of materials and methods can be found in Additional File 1. In brief, the following methods were used in this study: transcriptome data mining to identify differentially expressed genes, clinical HNSCC tissue cohort to validate candidate genes, cell culture models to validate gene expression and establish drug-resistant cell strains for functional analyses, cell viability assays to measure drug responses, pharmacological dose-response assays to identify drug-gene interactions, siRNA assays to validate candidate chemoresistant genes, reverse transcription quantitative PCR (RT-qPCR) to measure gene expression and drug library screens to identify potential existing known drugs to counteract HNSCC chemoresistance.

## Results

### Transcriptome data mining and gene selection

**Fig. 1:**
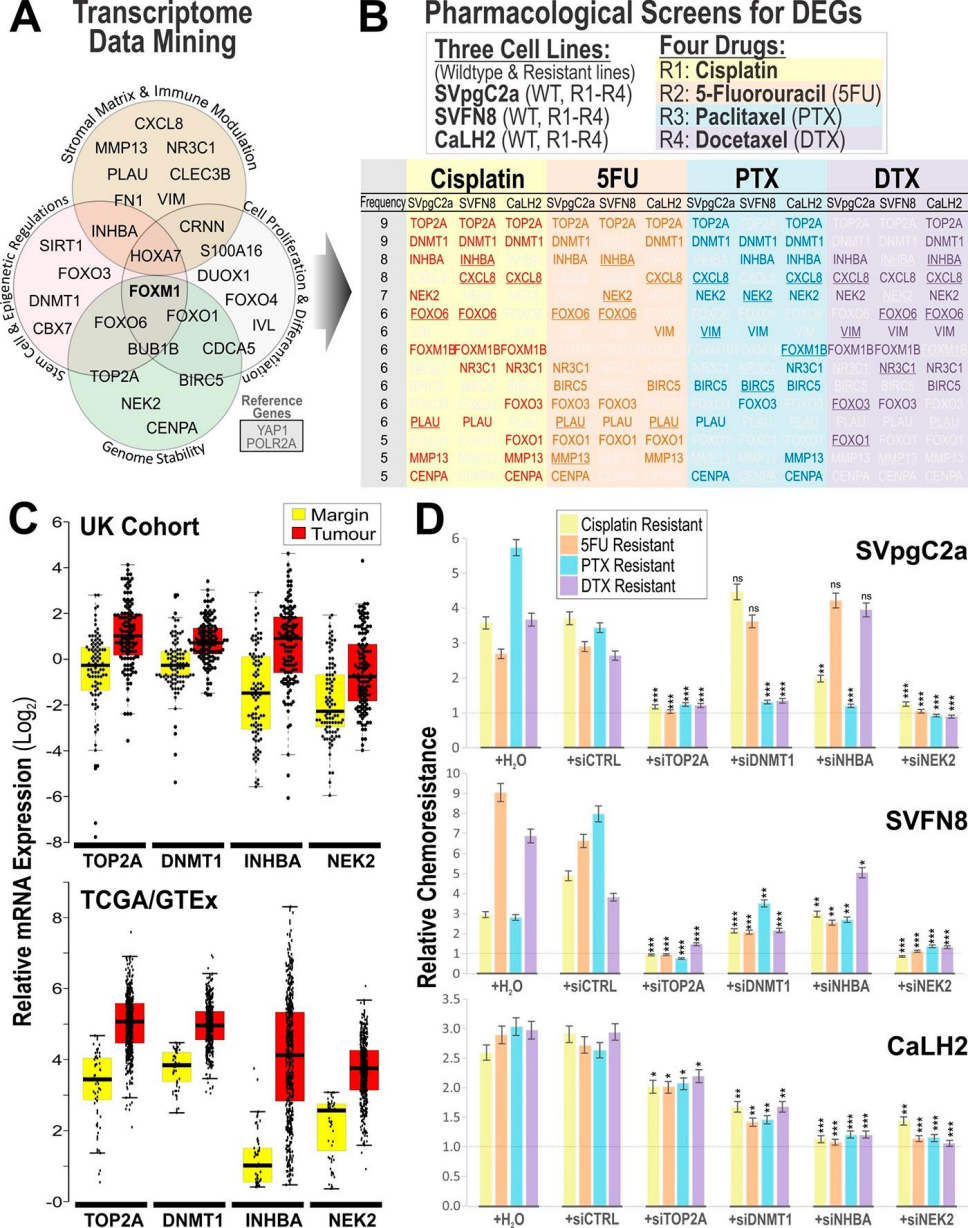
Identification and validation of candidate genes responsible for conferring multidrug resistance in HNSCC. **A**, Transcriptome data mining from eight independent gene expression microarray studies (comparing HNSCC tumour and normal oral tissues samples) identified 28 short-listed genes involved in the regulation of matrix remodelling, immune modulation, cell proliferation & differentiation, stem cell renewal, epigenetic programming and genomic instability. **B**, Pharmacological dose-response screening for multidrug-resistant differentially expressed genes in three cell lines (SVpgC2a, SVFN8 and CaLH2) each between parental WT cells and their corresponding drug-resistant strains for four chemotherapeutic drugs (R1: Cisplatin, R2: 5FU, R3: PTX and R4: DTX). For each cell/drug resistant combination strain, differential expression of the 28 genes were measured by RT-qPCR to identify drug dose-dependent response genes (see Additional File 2: Fig. S1-S12). Table shows the top differentially expressed genes in corresponding drug-resistant cell strains are shown in coloured (statistically significant) gene symbols (non-significant genes in white text). Genes with underlines indicate downregulation in drug-resistant cells, otherwise, upregulation. The list of 28 genes were ranked in descending order according to their frequency of occurrence as top significant genes across the whole panel of 12 cell/drug combinations. **C**, Validation of gene expression levels of TOP2A, DNMT1, INHBA and NEK2 in two different HNSCC patient cohorts: Top panel, a UK cohort with adjacent margin (n = 98) and HNSCC tumour core tissues (n = 123). The relative mRNA expression levels of each of the four genes were measured using RT-qPCR against two reference genes (YAP1 and POLR2A) measured in duplicate wells. Data were plotted as beeswarm dot-plot with box-and-whisker overlays (minimum, box: median, and 25–75%, percentiles and maximum). Statistical t-test were performed between the margin and tumour samples and all four genes showed P < 1 × 10^− 5^. Bottom panel: Differential expression of the four genes in HNSC cohort from TCGA/GTEx transcriptomic data comparing margin (n = 44) and HNSCC (n = 519) were all significantly upregulated in tumour (P < 0.01, one-way ANOVA). **D**, Effects of siRNA gene silencing of TOP2A, DNMT1, INHBA and NEK2 on reversal of chemoresistance (or re-sensitisation). Summary of relative chemoresistance following gene-specific siRNA knockdown in SVpgC2a, SVFN8 and CaLH2 cells, each resistant to either cisplatin, 5FU, PTX or DTX. Relative chemoresistance was calculated as fold-change between IC_50_ of drug-resistant cells and IC_50_ of corresponding WT cells. IC_50_ drug potency values of each of the four chemotherapeutic drugs on WT and drug-resistant cells were measured using crystal violet cell viability assay (Additional File 2: Fig. S14-S16). Statistical t-test was performed between controls (mock transfection/+H_2_O and siCTRL were combined as one group) vs. each of the gene-specific siRNA and their corresponding P-values are indicated (*<0.05; **<0.01; ***<0.001; ns, not significant) within the charts

Meta-analyses of eight independent HNSCC microarray studies (see Additional File 2: Table S1) were performed using the cancer microarray database Oncomine (www.oncomine.org) to identify differentially expressed genes in studies comparing HNSCC with normal oral mucosa. Initially, top 40 differentially expressed genes were selected based on their reported P-values (> 0.001). We performed RT-qPCR to quantify each of the 40 genes in a panel of eight primary normal human oral keratinocytes (OK355, HOKG, OK113, NOK, NOK1, NOK3, NOK16 and NOK376) and ten HNSCC cell lines (SCC4, SCC9, SCC15, SCC25, SqCC/Y1, UK1, VB6, CaLH2, CaDec12 and 5PT) to identify and validate differentially expressed genes. Of the 40 genes, 28 were found to be differentially expressed in our cell line panels and have been implicated in the regulation of matrix remodelling, immune modulation, cell proliferation & differentiation, stem cell renewal, epigenetic programming and genomic instability (Fig. 1A and Additional File 2: Table S2).

### Identification of common multidrug-resistant genes

With an aim to identify key genes that mediate chemoresistance in molecularly different background, we have selected three cell lines to represent diverse molecular background from oral premalignancy (SVpgC2a), carcinogen (nicotine)-transformed malignancy (SVFN8) and a patient HNSCC tumour-derived malignancy (CaLH2). In order to identify common multidrug-resistant genes, we generated four drug-resistant cell strains (R1: Cisplatin, R2: 5FU, R3: PTX and R4: DTX) for each of the three different cell lines (SVpgC2a, SVFN8 and CaLH2) giving rise to a total of 12 cell/drug-resistant combination strains (Fig. 1B). For each cell/drug-resistant combination strain, we challenged each wildtype (WT) and drug-resistant cell strain with the corresponding drug and measured the differential expression of the 28 genes by RT-qPCR to identify drug dose-dependent response genes to each chemotherapeutic drug (Additional File 2: Fig. S1-S12). We then performed statistical t-test and regression analyses on the 28 genes for each cell/drug-resistant strain to identify differentially expressed genes between drug-resistant and WT cells for each drug. To identify the most common drug-resistant genes, the 28 genes were ranked in descending order according to their frequency of occurrence as top significant genes across the whole panel of 12 cell/drug combinations. Of these, we selected the top four upregulated genes (TOP2A, DNMT1, INHBA and NEK2) across the entire cell/drug combinations to further investigate their roles in conferring multidrug resistance.

### Differential gene expression in HNSCC clinical tissue samples

In order to confirm that these four genes (TOP2A, DNMT1, INHBA and NEK2) were indeed upregulated in HNSCC tumours, we performed RT-qPCR on a UK HNSCC tissue cohort to quantify their relative gene expression levels in adjacent margin (n = 98) and HNSCC core tumour tissues (n = 123). All four genes were confirmed to be significantly upregulated in HNSCC tumour compared to margin tissues (P < 10^− 5^; Fig. 1C top panel). To cross validate our findings with an external cohort, we queried the four gene expression using the pan-cancer GEPIA ‘Box Plot’ tool based on transcriptomic data of The Cancer Genome Atlas (TCGA)/The Genotype-Tissue Expression (GTEx). In agreement, our findings are consistent with TCGA/GTEx HNSCC cohort demonstrating significant upregulation of all four genes in HNSCC (n = 519) over normal mucosa (n = 44) samples (P < 0.01; Fig. 1C bottom panel).

### Reversal of chemoresistance by siRNA gene silencing

To investigate if the four genes (TOP2A, DNMT1, INHBA and NEK2) identified above were conferring multidrug resistance across the 12 different cell strains, we performed gene silencing using siRNA to knockdown each of these genes in both the WT and drug-resistant cells in response to each corresponding drug. We hypothesised if the genes were necessary to sustain drug resistance, knockdown of the genes would abrogate chemoresistance. To test this hypothesis, we transfected gene-specific siRNA and treated both WT and drug-resistant cells to serial-dilutions of corresponding drug to determine their IC_50_ values (drug potency). Abrogation of chemoresistance would result in a shift in IC_50_ values of resistant cells towards IC_50_ of WT cells (i.e., reducing the fold difference between the two IC_50_ values). We included untransfected (+ H_2_O, containing transfection reagent only) and control siRNA (siCTRL) as controls and confirmed gene-specific siRNA silencing by RT-qPCR (Additional File 2: Fig. S13). We screened for reversal of chemoresistance by siRNA against each of the four genes in the three cell lines (SVpgC2a, SVFN8 and CaLH2) each with drug-resistance to each of the four chemotherapeutic drugs (cisplatin, 5FU, PTX and DTX). The chemosensitivity (IC_50_-fold change between resistant and WT cells) of all the 12 cell strains are summarised in Fig. 1D and individual dose-response curves data are shown in Additional File 2: Fig. S14-S16. Gene expression levels and IC_50_ values in siCTRL transfected cells were very similar to untransfected cells indicating that siCTRL did not induce any non-specific or off-target effects. In SVpgC2a cells, siTOP2A and siNEK2 both completely reversed chemoresistance (P < 0.001) in all four drug-resistant strains. siDNMT1 reversed only PTX- and DTX-resistant strains whilst, siINHBA reversed only cisplatin and PTX-resistant strains. In SVFN8 cells, similar to results from SVpgC2a cells, siTOP2A and siNEK2 completely reversed drug resistance in all four drug-resistant strains. However, unlike results for SVpgC2a, siDNMT1 and siINHBA showed partial reversal of resistance in all four different drug-resistant strains. Interestingly, in CaLH2 cells (HNSCC tumour-derived cell line), siNEK2 and siINHBA showed complete reversal of drug resistance in all four drug-resistant strains, whilst siDNMT1 and siTOP2A showed only partial reversal of chemoresistance.

### Pan-cancer Kaplan-Meier prognostic biomarker meta-analysis

**Fig. 2:**
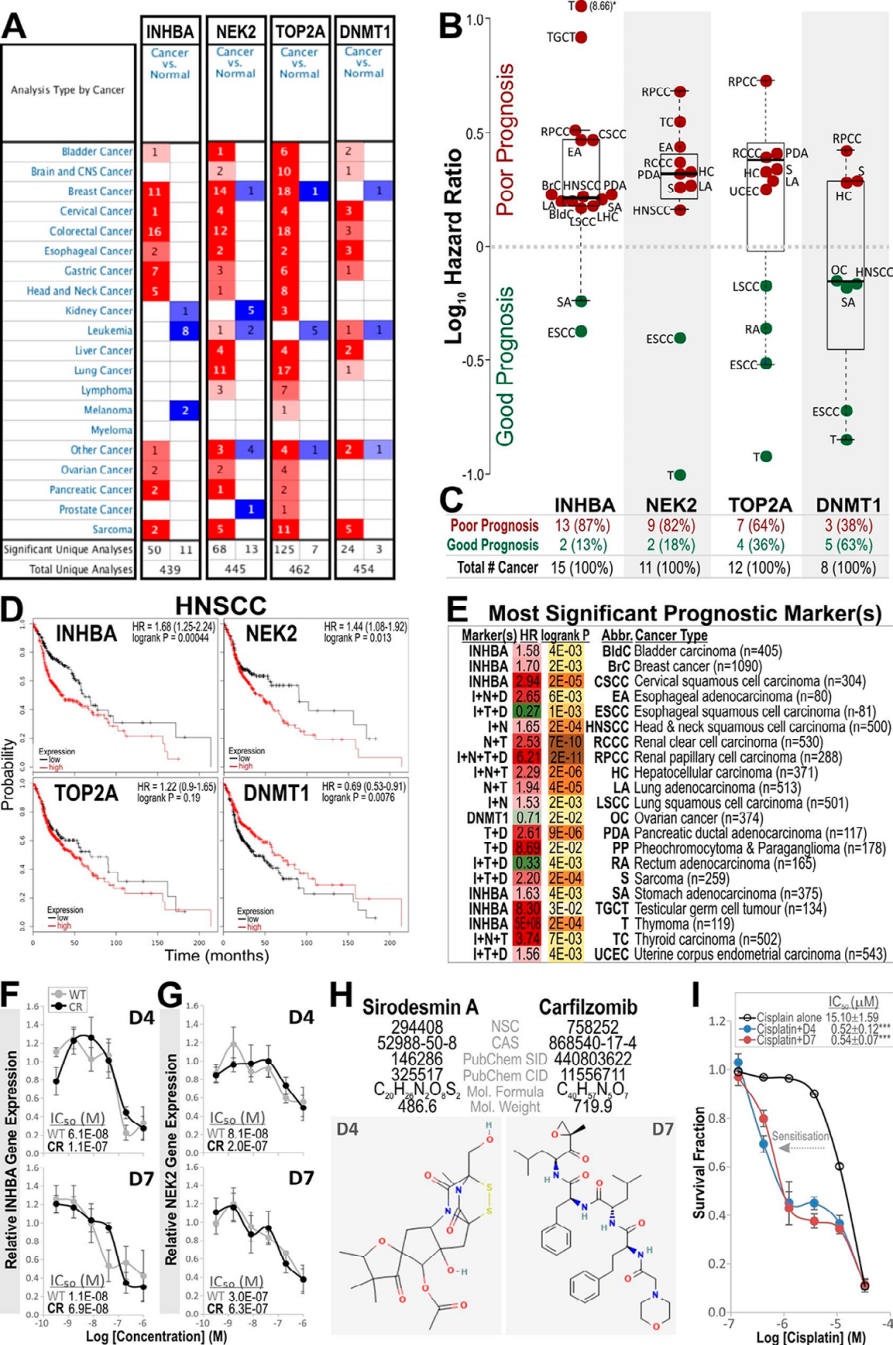
Pan-cancer bioinformatics data mining and prognostic analysis. **A**, Bioinformatics data mining from Oncomine databases on differential gene expressions of INHBA, NEK2, TOP2A and DNMT1 across 20 different human cancer types as indicated. The number within each coloured box indicates the number of significant unique studies. Red and blue colours indicate gene expression upregulation and downregulation, respectively. Cell colour scale is determined by the best gene rank percentile for the analyses (dark red/blue = top 1%; red/blue = top 5%; pale red/blue = top 10%). **B**, Kaplan-Meier RNA-seq transcriptome prognostic analysis for INHBA, NEK2, TOP2A and DNMT1 on 21 different human cancer types. Hazard ratios (with logrank P < 0.05) extracted from KM-plotter database were plotted here as beeswarm dot-plot with box-and-whisker overlays (minimum, box: median, and 25–75%, percentiles and maximum) to demonstrate individual marker prognostic value for each cancer type. Dark red indicates marker associated with poor prognosis and green for markers associated with good prognosis (abbreviations listed in panel E). *Note: outlier (Thymoma, Log_10_ HR = 8.66) was plotted outside the chart for reference. **C**, Table listing corresponding number (and %) of cancer type analysed for each marker with poor or good prognosis. **D**, Individual Kaplan-Meier plots for INHBA, NEK2, TOP2A and DNMT1 in HNSCC tumour samples (n = 500) with hazard ratio (HR) and logrank P values as shown within each panel. **E**, All four markers (alone and in combinations) were examined for their synergistic prognostic values for each cancer type, the most significant prognostic marker (or markers in combinations) are tabulated here. Hazard ratio (HR) values were shown with colour scales applied to indicate poor (dark red) or good (green) prognosis with their corresponding logrank P values (colour scales indicate their relative levels of significance) for each cancer type (n = the number of samples in each cancer type). Marker abbreviations: I, INHBA; N, NEK2; T, TOP2A and D, DNMT1). **F-I**, Drug library screen to identify drug-gene interactions for counteracting chemoresistance in HNSCC cells. Nine compounds (D1-D9; Additional File 2: Fig. 17) were selected. Shown here are two compounds (D4, and D7) with dose-dependent inhibition on both INHBA (**F**) and NEK2 (**G**) gene expression in WT and CR CaLH2 cells. Each datapoint represents relative gene expression (mean ± SEM) of quadruplicates quantified using RT-qPCR. Drug potencies (IC_50_) on respective gene inhibition are displayed within each panel. **H**, Chemical structure and identity of compounds D4 and D7. **I**, Re-sensitisation of CR CalH2 cells by addition of D4 or D7 (1 µM) to cisplatin dose-response measured using AlamarBlue cell viability assay. Each datapoint represents a mean ± SEM of n = 6 replicates. Cisplatin potency values (mean IC_50_ ± SEM of n = 6) in the absence or presence of either D4 or D7 single concentration (1 µM) are shown within the figure. Statistical t-test was performed between cisplatin alone vs. cisplatin + D4 or cisplatin + D7 and their corresponding P-values are indicated within the figure as ***<0.001.

To further investigate the four genes (TOP2A, DNMT1, INHBA and NEK2) differential expression and if they have any prognostic value in different human cancer types, we performed data mining on publicly available pan-cancer databases Oncomine and KM Plotter with RNA-seq transcriptome (http://km-plotter.com/) containing 54,675 genes with survival outcome for 21 different human cancer types (Additional File 3). All four genes were found to be upregulated in the majority of human cancer types with few minor exceptions (Fig. 2A). Upregulation of INHBA, NEK2, TOP2A and DNMT1 were associated with poor prognosis in 13 (87%), 9 (82%), 7 (64%) and 3 (38%) of tumour types, respectively (Fig. 2B C). For HNSCC, upregulation of INHBA, NEK2 and TOP2A but not DNMT1 were associated with poor prognosis (Fig. 2D). All four marker (alone and in combinations) were examined for their synergistic prognostic values for each cancer type and the most significant prognostic marker (or markers in combinations) are shown in Fig. 2E. Overall, INHBA followed by NEK2 appeared to be pan-cancer prognostic markers for predicting poor survival outcome in the majority of cancer types. INHBA (alone and/or in combinations with TOP2A, NEK2 or DNMT1) predicted poor prognosis in 16 out of 21 different human cancer types, including HNSCCs (Fig. 2E). These data are consistent with data found in another pan-cancer database Gene Expression Profiling Interactive Analysis (GEPIA) based on the Cancer Genome Atlas (TCGA) and Genotype-Tissue Expression (GTEx) RNA-seq data for at least 33 human cancer types (Additional File 1).

### Identification and repurposing drugs targeting INHBA and NEK2 for counteracting cisplatin resistance

We performed a cell viability drug screen on a total of 537 compounds consisting of 147 approved oncology drugs (AOD IX) and 390 natural products (Set V) in WT and cisplatin-resistant (CR) CaLH2 with an aim to identify and repurpose existing drugs that suppress INHBA and/or NEK2 gene expression to counteract chemoresistance in HNSCC. We chose the CaLH2 cell line to investigate the effect of candidate drugs as both INHBA and NEK2 genes were not found to be differentially expressed between WT and CR cells in respond to cisplatin (Fig. 1B). The initial viability screening results led us to select nine most effective compounds (P < 0.05), of which three compounds (D1-D3) were selected as control drugs with specificity for killing WT but not CR cells. The next three compounds (D4-D6) were specific for killing CR cells and the remaining three compounds (D7-D9) killed both WT and CR cells (Additional File 2: Fig. 17A). To investigate if the nine compounds were capable of inhibiting INHBA and NEK2 gene expression in a dose-dependent manner in respective WT and CR cells, we treated cells with serial dilution of each of the nine compounds and measured relative gene expression levels of INHBA and NEK2 using RT-qPCR. The first three control compounds (D1-D3), consistent with cell viability results, showed dose-dependent inhibition on both INHBA and NEK2 expression in WT cells but not in CR cells. Of the remaining drugs, D4 and D7 dose-dependently inhibited with comparable potency on both INHBA (Fig. 2F) and NEK2 (Fig. 2G) gene expression in both WT and CR cells. D5 inhibited only INHBA but not NEK2. D9 did not show dose-dependent gene inhibition in either WT or CR cells (Additional File 2: Figure 17B-C). Potency (IC_50_) of D4, D5 and D7 on gene inhibition were found to range from 1.1 × 10^− 8^ M to 6.3 × 10^− 7^. As D5 only inhibited INHBA and not NEK2, this compound was not further investigated. The chemical structures and identities of the two selected compounds D4 (Sirodesmin A) and D7 (Carfilzomib) are shown in Fig. 2H (ID of other compounds are shown in additional File 2: Fig. 17D). Subsequent cisplatin dose-dependent cell viability assays in the presence of a single dose (1 µM) of either D4 or D7 re-sensitised (leftward shift in dose-respond curves) the potency (IC_50_) of cisplatin from 15.10 ± 1.59 µM to 0.52 ± 0.12 µM (29-fold by D4; t-test P = 1.7 × 10^− 5^) or to 0.54 ± 0.07 µM (27.7-fold by D7; P = 3.6 × 10^− 6^), respectively. This demonstrated that cisplatin-resistant cells could be significantly re-sensitised to cisplatin by addition of either D4 or D7 (Fig. 2I). We noted a biphasic cell viability cisplatin dose-response curves in the presence of either D4 or D7 (1 µM); this may indicate the involvement of multiple mechanisms of cisplatin resistance. Both D4 or D7 showed partial dose-dependent sensitisation only within the lower doses (0.1-1 µM) of cisplatin but plateaued in higher doses (1–10 µM) of cisplatin. We speculated that this could be due to the presence of different populations of cells (e.g., EMT cells and stem cells) within the culture and/or that there are multiple distinct signalling pathways involved. D4 and D7 could be acting only on one of these cisplatin-resistant pathways, hence demonstrating a biphasic response. Further investigation is required to delineate these mechanisms.

## Discussion

Currently there are no specific molecular biomarkers that can indicate which HNSCC patient is susceptible to developing drug resistance. This study presented a series of experiments using a combination of bioinformatics data mining, cell culture model and pharmacological approaches to identify key transcriptome biomarkers that confer multidrug chemoresistance. We have identified ten candidate genes (TOP2A, DNMT1, INHBA, CXCL8, NEK2, FOXO6, VIM, FOXM1B, NR3C1 and BIRC5) of which, four (TOP2A, DNMT1, INHBA and NEK2) were confirmed to be significantly upregulated in our UK HNSCC tumour cohort and also consistent with TCGA/GTEx data. NEK2, DNMT1 and FOXM1B had been previously shown to be upregulated in HNSCC tumour cohorts from UK, Norway and China [5, 6]. Furthermore, TOP2A, DNMT1, INHBA, CXCL8, NEK2, NR3C1 and BIRC5 were previously shown to be differentially expressed in independent HNSCC patient cohorts from UK, China and India [7], and these genes were part of a multigene biomarker panel for molecular diagnosis of HNSCC and risk stratification in dysplastic oral premalignant disorders [7]. Nevertheless, their roles in HNSCC chemoresistance remain unclear.

Here, we uncovered an essential role for a cell cycle gene, NEK2 (Never in mitosis gene A-related kinase 2), in conferring multidrug chemoresistance in HNSCC cells whereby targeted siRNA gene silencing against NEK2 led to complete abrogation of chemoresistance in all 12 chemoresistant cell strains (to four different drugs: cisplatin, 5FU, PTX and DTX). Consistently, our pan-cancer Kaplan-Meier survival analysis showed that upregulation of NEK2 predicted poor prognosis in HNSCC patients. Although NEK2 has been previously reported to confer chemoresistance in multiple human malignancies, to our knowledge we presented the first evidence for NEK2 in conferring multidrug chemoresistance in HNSCC. We have also presented evidence for INHBA (inhibin subunit beta A), TOP2A (DNA topoisomerase II alpha) and DNMT1 (DNA methyltransferase 1) in conferring chemoresistance in the majority of drug-resistant cell strains. Targeted siRNA on each of these genes showed mixed responses across the 12 chemoresistance cell strains perhaps due to inherent heterogeneity of the different parental cell lines. Further investigation is required to delineate and differentiate their molecular pathways in these cell strains.

In support of our HNSCC cohort data, previous studies have also demonstrated differential upregulation of INHBA in HNSCC [8]. Our pan-cancer Kaplan-Meier survival analysis further revealed that INHBA (alone and/or in combinations with TOP2A, NEK2 or DNMT1) predicted poor prognosis in 16 out of 21 different human cancer types, including HNSCC. In agreement with a role in chemoresistance found in this study, INHBA has been shown to be part of a 7-gene prognostic signature that predicts the outcome of HNSCC patients treated with postoperative radio(chemo)therapy [9]. Given that INHBA gene encodes a member of the TGF-beta (transforming growth factor-beta) superfamily of proteins which involve in the regulations of EGFR (epidermal growth factor receptor) [10] and oncogenic transcription factor RUNX2 [11] pathways in HNSCC cells, highlights the significance of INHBA as an important novel molecular target and prognostic biomarker for HNSCC.

With an aim to repurpose the use of licensed drugs for counteracting cisplatin resistance in HNSCC, from our drug-gene interaction library screens, we identified two drugs (Sirodesmin A and Carfilzomib) targeted both INHBA and NEK2 in a dose-dependent manner and re-sensitised cisplatin resistant cells. Sirodesmin A is a natural metabolite produced by the fungus Sirodesmium diversum (ascomycete fungi) [12] and little is known about its activity on human cancer cells. To our knowledge, we presented the first evidence that Sirodesmin A counteracted cisplatin resistance in a HNSCC cell line and dose-dependently inhibited both INHBA and NEK2 gene expression. Carfilozomib (Kyprolis®), a derivative of a bacterial actinomycete irreversible proteosome inhibitor epoxomicin, is licensed for treating patients with relapsed and/or refractory multiple myeloma [13]. Whilst Carfilozomib has been shown to potentiate the effect of cisplatin in a number of cancer types such as multiple myeloma [14], ovarian [15] and neuroblastoma [16], our results demonstrated for the first time that Carfilozomib dose-dependently inhibited both INHBA and NEK2 gene expression, and re-sensitise cisplatin resistant HNSCC cells. We hypothesised that Sirodesmin A or Carfilozomib could be repurposed to counteract cisplatin resistance in tumours with elevated NEK2 and/or INHBA gene expression. Further investigations are necessary to delineate their drug-gene interactions and mechanism of actions in counteracting chemoresistance.

## Conclusions

We presented the first evidence for NEK2 in conferring multidrug chemoresistance in HNSCC cells and that targeted siRNA gene silencing led to complete reversal of drug resistance to cisplatin, 5FU, PTX and DTX. INHBA and TOP2A were found to confer chemoresistance in the majority of drug-resistant cell strains whereas DNMT1 showed heterogeneous effects on chemoresistance. Pan-cancer Kaplan-Meier survival analysis on 21 human cancer types revealed significant prognostic values for NEK2 and INHBA in the majority of cancer types. We further identified a naturally occurring fungal derivative Sirodesmin A and a licensed anticancer drug Carfilzomib, both targeting NEK2 and INHBA, could re-sensitise resistant HNSCC cells to cisplatin. This finding requires further investigations into the potential of repurposing licensed drugs for reversing chemoresistance in HNSCC patients.

### Electronic supplementary material

Below is the link to the electronic supplementary material.

Supplementary Material 1

Supplementary Material 2

Supplementary Material 3

## Data Availability

“All data generated or analysed during this study are included in this published article [and its supplementary information files]”.

## Abbreviations

5FU: 5-fluorouracil
cDNA: complementary DNA
CR: cisplatin resistant cells
DMEM: Dulbecco’s Modified Eagle Medium
DNMT1: DNA methyltransferase 1
DTX: docetaxel
FOXM1B: forkhead box M1 isoform B
IC_50_: Drug potency, concentration of drug which induced 50% inhibition
HNOK: human normal oral keratinocytes
HNSCC: head and neck squamous cell carcinoma
HPV: human papilloma virus
INHBA: inhibin subunit beta A
KM: Kaplan-Meier
miRNA: micro-RNA
NEK2: Never in mitosis gene A-related kinase 2
NHS: National Health Service
PD-1: programmed cell death 1
PTX: paclitaxel
QMUL: Queen Mary University of London
RT: room temperature
RT-qPCR: reverse transcription quantitative polymerase chain reaction
SCC: squamous cell carcinoma
siRNA: short interfering RNA
SV40T: Simian virus 40T
SVFN8: a transformed/malignant cell line derived from SVpgC2a
SVpgC2a: premalignant SV40T-antigen immortalised human buccal keratinocytes
TGF-beta: transforming growth factor-beta
TOP2A: DNA topoisomerase II alpha
WT: wildtype cells
