# Supplementary material for: Identification of multidrug chemoresistant genes in head and neck squamous cell carcinoma cells
Source: Mol Cancer. 2023 Sep 4;22:146. doi: 10.1186/s12943-023-01846-3 (PMC10476423; doi:10.1186/s12943-023-01846-3)
Supplement: Supplementary file 1 — Supplementary Material 1 [file 12943_2023_1846_MOESM1_ESM.pdf]

Materials and Methods

Clinical Samples

The use of fresh clinical specimens collected in the UK was approved by the NHS Research Ethics Committee (06/MRE03/69). All tissue samples were previously collected according to local ethical committee-approved protocols and informed patient consent was obtained from all participants [1-3]. Fresh tissue biopsies were preserved in RNALater (#AM7022, Ambion, Applied Biosystems, Warrington, UK) and stored short-term at 4°C (1-7 days) prior to transportation and subsequent storage at -20°C until used. All frozen samples were digested with nuclease-free proteinase K at 60°C prior to mRNA extraction (Dynabeads mRNA Direct kit, Invitrogen).

Cell Culture & Establishment of Drug-resistant Cells

All cell lines were cultured in Dulbecco's Modified Eagle Medium (DMEM) with 10% foetal bovine serum and 1% penicillin/streptomycin and maintained in a humidified incubator with 5% CO_2/95% atmospheric air at 37°C. All primary normal human oral keratinocytes (OK355, HOKG, OK113, NOK, NOK1, NOK3, NOK16 and NOK376) were extracted from normal oral mucosa donated by healthy disease-free individuals undergoing wisdom tooth extraction and cultured as previously described [4, 5]. SVpgC2a was a non-transforming/premalignant Simian virus 40T (SV40T)-antigen immortalised human buccal keratinocytes [6] and SVFN8 was a carcinogen (nicotine) transformed/malignant cell line derived from SVpgC2a [4]. HNSCC cell lines (SCC4[7], SCC9[7], SCC15[7], SCC25[7], SqCC/Y1[8], UK1[9], VB6[9], CaLH2[9], CaDec12[9] and 5PT[9]) are all well characterised lines and were cultured as described previously [4, 5, 10]. The p53 mutational status of the three cell lines (SVpgC2a, SVFN8 and CaLH2) used in the current study for generating chemoresistant lines is not known. However, according to our previous transcriptome profiling data (NCBI's GEO database GSE89217 [11]), all these three cell lines showed undetectable level of p53 gene expression. Several mechanisms including gene silencing, gene deletion, mutations that lead to premature stop codon, etc., could contribute to undetectable p53 mRNA levels. As these are beyond the scope of the current study, future sequencing experiments would need to be conducted to identify the type of mutations involved in these cell lines.

Crystal Violet Cell Viability Assay

Crystal violet cell viability assay was performed in 96-well plates (Figure S1). Growth medium was aspirated and 30 µL/well of crystal violet solution (0.5% crystal violet in 30% ethanol) was added and incubated for 10 minutes at room temperature (RT). Cells were then washed with 200 µL/well distilled water prior to the addition of 100 µL/well of 1% SDS and incubated for 30 minutes at RT prior to absorbance measurement at 595 nm using a CLARIOstar microplate reader.

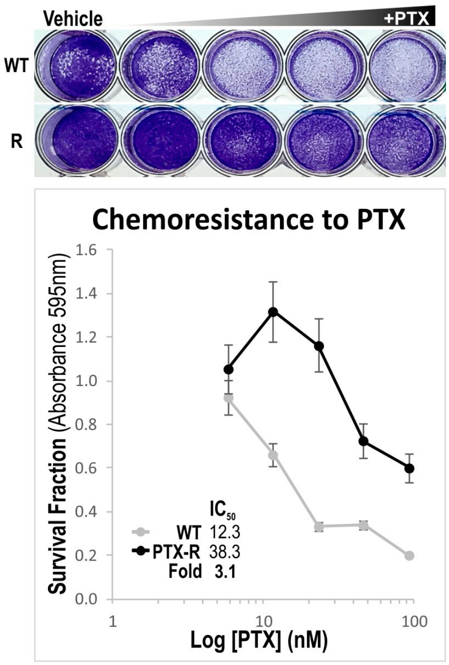

Figure S1. *Chemosensitivity assays for wildtype (WT) and drug-resistant cells measured by crystal violet cell viability assay following 72 hr drug incubation. An example shown here using WT and PTX-resistant (PTX-R) CaLH2 cell lines.* IC_{50} *values (shown here in nM) representing the degree of chemosensitivity for WT and PTX-R cells were determined using sigmoid-curve fitting algorithm on respective data points plotted as logarithmic drug concentrations on the X-axis and survival fraction (Absorbance at 595 nm) on the Y axis.* IC_{50}*-fold difference was calculated between WT and PTX-R cells as indicated within the graph.*

Establishment of Drug-resistant Cell Strains

Crystal violet cell viability dose-response curve (kill-curve) assays were first performed to determine the half maximal growth inhibition (IC_{50}) concentrations of cisplatin, 5-fluorouracil (5FU), paclitaxel (PTX) and docetaxel (DTX) on SVpgC2a, SVFN8 and CaLH2 cell lines. Each cell type was then cultured in growth media containing IC_{50} concentrations of each drug with regular changes of growth media containing freshly diluted drugs. When cells were proliferating, a 3-fold higher concentration above the IC_{50} of each drug were then added to the growth medium. This process was repeated until cells were able to proliferate in the highest drug concentrations over a period of ~6 months. Drug-resistant cells were then expanded in drug-free growth medium to create aliquots for cryopreservation until used for experiments. We have established a total of 12 drug resistant strains: four drug-resistant (Cisplatin, 5FU, PTX and DTX) strains for each of the three cell types (SVpgC2a, SVFN8 and CaLH2), with a minimum of 3-fold higher IC_{50} values than their corresponding parental wildtype cells Additional File 2: Figure S14-S16).

Drug-dependent Chemoresistant Gene Expression Assay

To identify genes that are differentially expressed when challenged with increasing doses of chemotherapeutic drugs in WT and its corresponding drug-resistant strains, cells were seeded into 96-well plate (8,000 cells/well) one day prior to drug treatment. Cells were then treated with serial dilutions of each drug for 24h prior to harvest for RT-qPCR to quantify the relative mRNA expression levels of 28 target genes (Figure S2).

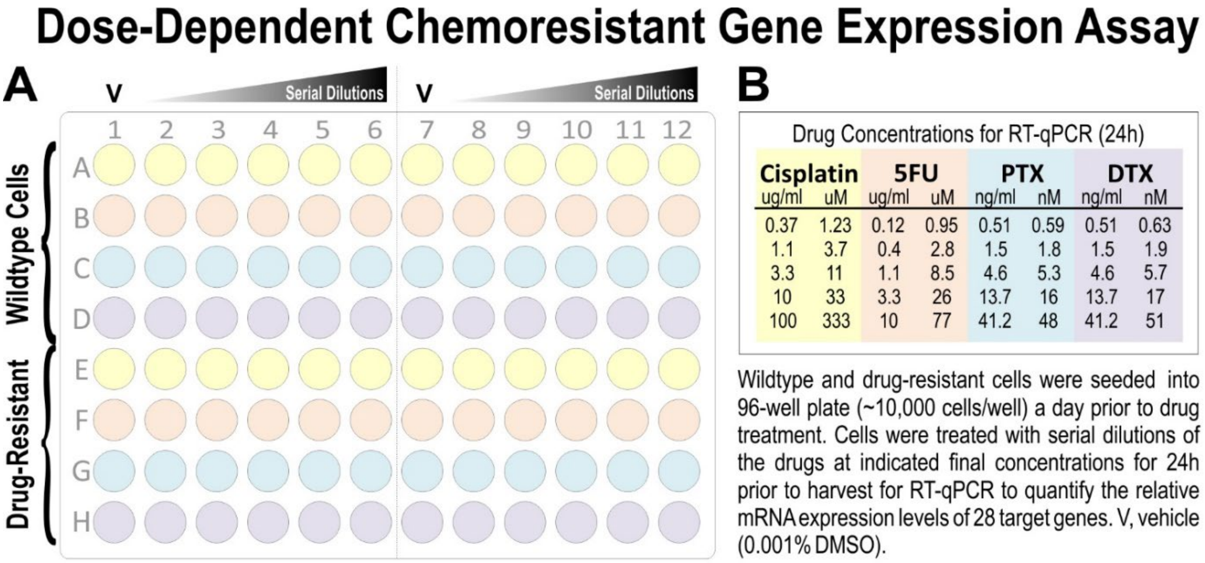

Figure S2. *Dose-dependent chemoresistant gene expression assay on wildtype and drug-resistant cells treated with varying doses of corresponding drugs as indicated. A, 96-well plate assay format. B, Final drug serial-concentrations used for each drug.*

Reverse transcription quantitative PCR (RT-qPCR)

RT-qPCR assays were performed as described previously [1-3] with minor modifications. Briefly, mRNA purified directly from cells using the Dynabeads™ mRNA DIRECT™ Purification Kit (61012; ThermoFisher Scientific, UK) were used directly in RT-qPCR reaction containing qPCRBIO SyGrene 1-Step Go Lo-ROX (PB25.31-12; PCRBiosystems, UK) and gene-specific primers for one-step reverse transcription and qPCR to quantify gene expression in the LightCycler 480 qPCR system (Roche, UK) based on our previously published protocols [1, 3, 11] which are MIQE compliant [12]. Briefly, thermocycling begins with 45ºC for 10 mins (for reverse transcription) followed

by 95ºC for 30s prior to 45 cycles of amplification at 95ºC for 1s, 60ºC for 1s, 72ºC for 1s, 78ºC for 1s (data acquisition). A 'touch-down' annealing temperature intervention (66ºC starting temperature with a stepwise reduction of 0.6ºC/cycle; 8 cycles) was introduced prior to the amplification step to maximise primer specificity. Melting analysis (95ºC for 30s, 75ºC for 30s, 75-99ºC at a ramp rate of 0.57ºC/s) was performed at the end of qPCR amplification to validate single product amplification in each well. Relative quantification of mRNA transcripts was calculated based on the second derivative maximum algorithm [13] (Roche). Primer sequences are provided in Additional File 2: Table S2. All target genes were normalised to two stable reference genes validated previously [4] to be amongst the most stable reference genes across a wide variety of primary human epithelial cells, dysplastic and squamous carcinoma cell lines, using the GeNorm algorithm [14]. No template controls (NTCs) were prepared by omitting cells/tissue sample during RNA purification and eluates were used as NTCs for qPCR assays to monitor contamination.

siRNA gene silencing on reversal of chemoresistance

WT and drug-resistant cells were transfected with ON-TARGETplus SMARTpool siRNA (Horizon Dharmacon) for human NEK2 (L-004090-00-0005), DNMT1 (L-004605-00-0005), INHBA (L-011701-00-0005), TOP2A (L-004239-00-0005) or Non-targeting Pool siCTRL (D-001810-10-05) using DharmaFECT 1 transfection reagent (T-2001-02) according to DharmaFECTTM reverse transfection protocol provided by Horizon Dharmacon. Briefly, cells were seeded into 96-well plate (~5,000 cells/well) containing corresponding serial dilution of each drug (at 100x concentration) and siRNA transfection mix (containing siRNA in 0.22 µL/well of DharmaFect 1 transfection reagent) to provide a final siRNA concentration of 55 nM/well. Control cells received the same concentration of transfection reagent but without siRNA added. Details of drug concentrations, 96-well plate assay setup and siRNA reverse transfection protocol are shown in Figure S3 below. Following 3-day incubation, cells were harvested for RT-qPCR to confirm specific siRNA gene knockdown (Additional File 2: Figure S13) and crystal violet cell viability assay to investigate siRNA-induced reversal of chemoresistance (Additional File 2: Figure S14-16).

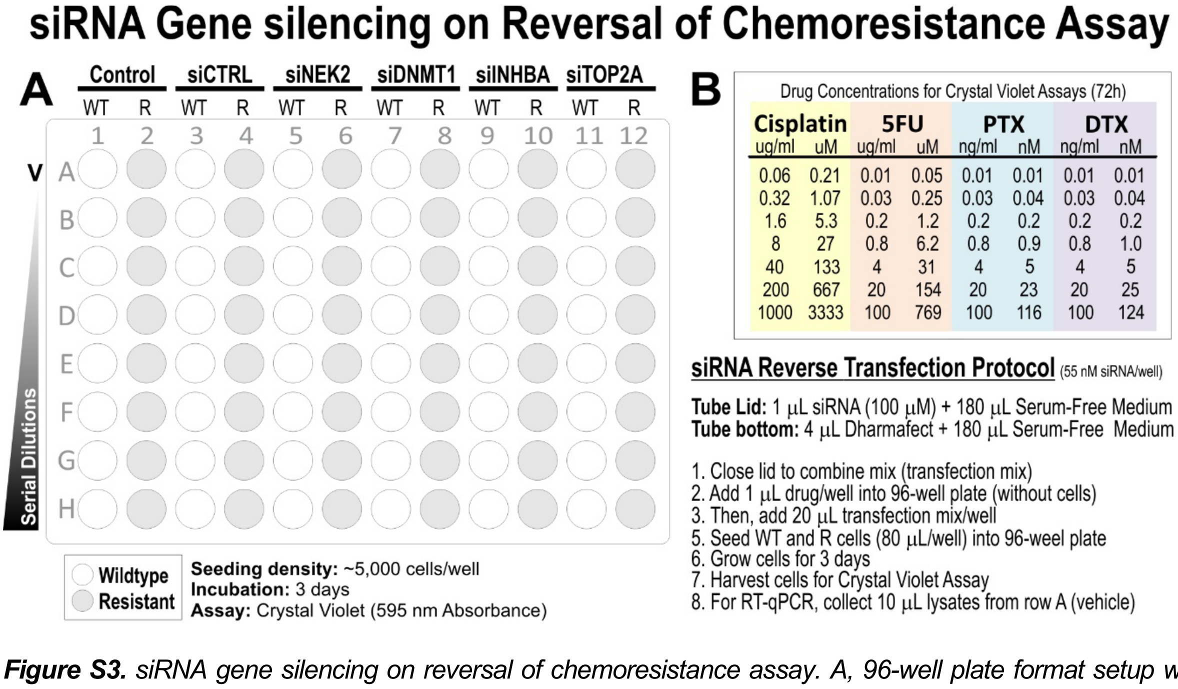

Figure S3. *siRNA gene silencing on reversal of chemoresistance assay. A, 96-well plate format setup with wildtype (WT) and drug-resistant (R) cells were seeded in wells containing corresponding final concentrations of serially diluted drugs (shown in B) and in the presence of corresponding siRNA (55nM final concentration per well) transfection mix.*

Determination of Chemosensitivity (IC_{50})

Chemosensitivity or drug potency on cell viability or gene expression was determined by performing curve-fitting on dose-response data points to calculate the concentration of drug which induced 50% inhibition (IC_{50}) using the four-parameter logistic Hill equation [15]:

$$Y = \frac{A-D}{1+\left(\frac{X}{C}\right)^{B}} + D$$

where Y is the percentage of cell death or gene downregulation, X is the drug concentration, A is the maximal cell density or gene expression, B is the slope factor, C is the IC_{50} and D is the minimal cell density or gene level. Cell viability or gene expression datapoints of dose-response assays were curve-fitted based on the above algorithm using the Quest Graph™ IC_{50} Calculator (AAT Bioquest, Inc, 04 Jul. 2019, https://www.aatbio.com/tools/ic50-calculator).

Pan-cancer and HNSCC transcriptome data mining

Pan-cancer transcriptome datasets were queried in the Oncomine (www.oncomine.org) [16] and Kaplan-Meier Plotter (KM-Plotter.com) [17] databases. The initial differentially expressed gene selection study was performed in Oncomine with the main inclusion criterion that the studies must involve comparison between HNSCC tumour samples with normal tissues. Studies using HNSCC cell lines were excluded. At the time of analysis, there were eight studies eligible for analysis (Additional File 2: Table S1). Differentially expressed genes were ranked according to their median P-values for over-expression and under-expression. Candidate genes were selected based on their top-ranking positions across the eight studies. For differential gene expression of selected candidate genes in HNSCC tumour tissues and matching normal margins, we have used the pan-cancer GEPIA 'Box Plot' tool (GEPIA, http://gepia.cancer-pku.cn/) [18] which is based on transcriptomic data of The Cancer Genome Atlas (TCGA)/The Genotype-Tissue Expression (GTEx) [19]). We have also used GEPIA to survey candidate gene expression profile across 33 human cancer types. For pan-cancer biomarker prognostic survival analysis, hazard ratio (HR) and logrank P values were extracted from each corresponding Kaplan-Meier plots for each cancer type with either single marker or different combinations of 2, 3 or 4 markers (there were a total of 15 unique combinations of 1 to 4 markers studied). The main exclusion criterion was when HR values were associated with logrank P values of <0.05 (Additional File 3).

Drug Library Screen

A total of 537 compounds were obtained from the Drug Synthesis & Chemistry Branch, Developmental Therapeutics Program (DTP) at the National Cancer Institute (National Institute of Health, USA), consisting of 147 approved oncology drugs (AOD IX) and 390 known natural products (Set V) selected from the DTP Open Repository collection of 140,000 compounds. Factors in selection were origin, purity (>90% by ELSD, major peak has correct mass ion), structural diversity and availability of compound. Drug library compounds were each given individual NSC ID number searchable at DTP Chemical database (https://dtp.cancer.gov/dtpstandard/ChemData/index.jsp). Original drug stocks (10 mM in DMSO) were diluted to 0.1 mM (in DMSO) as working stocks arrayed in 384-well reservoir plates for downstream screening using alamarBlue™ Cell Viability Reagent (DAL1025/DAL1100; ThermoFisher Scientific, Paisley, UK) in 384-well format. Cells (4000 cells/well in 384-well plates) were seeded one day before the addition of drugs (final drug concentration at 1 µM) which were incubated for 72h before addition of alamarBlue™ for 24h incubation before measuring fluorescence (excitation 540 nm and emission 590 nm) using a CLARIOstar microplate reader. Candidate drugs were selected based on anti-proliferative effects between wildtype and drug-resistant cells. For drug-gene dose-dependent interaction study, cells (8000 cells/well in 96-well plates) were incubated with candidate drugs (5-fold dilution containing 6 concentrations from 0.32 nM to 1 µM) for 24h prior to harvest for RT-qPCR to investigate their dose-response effects on gene expression. Assay format and detail protocol are shown in Figure S4 below.

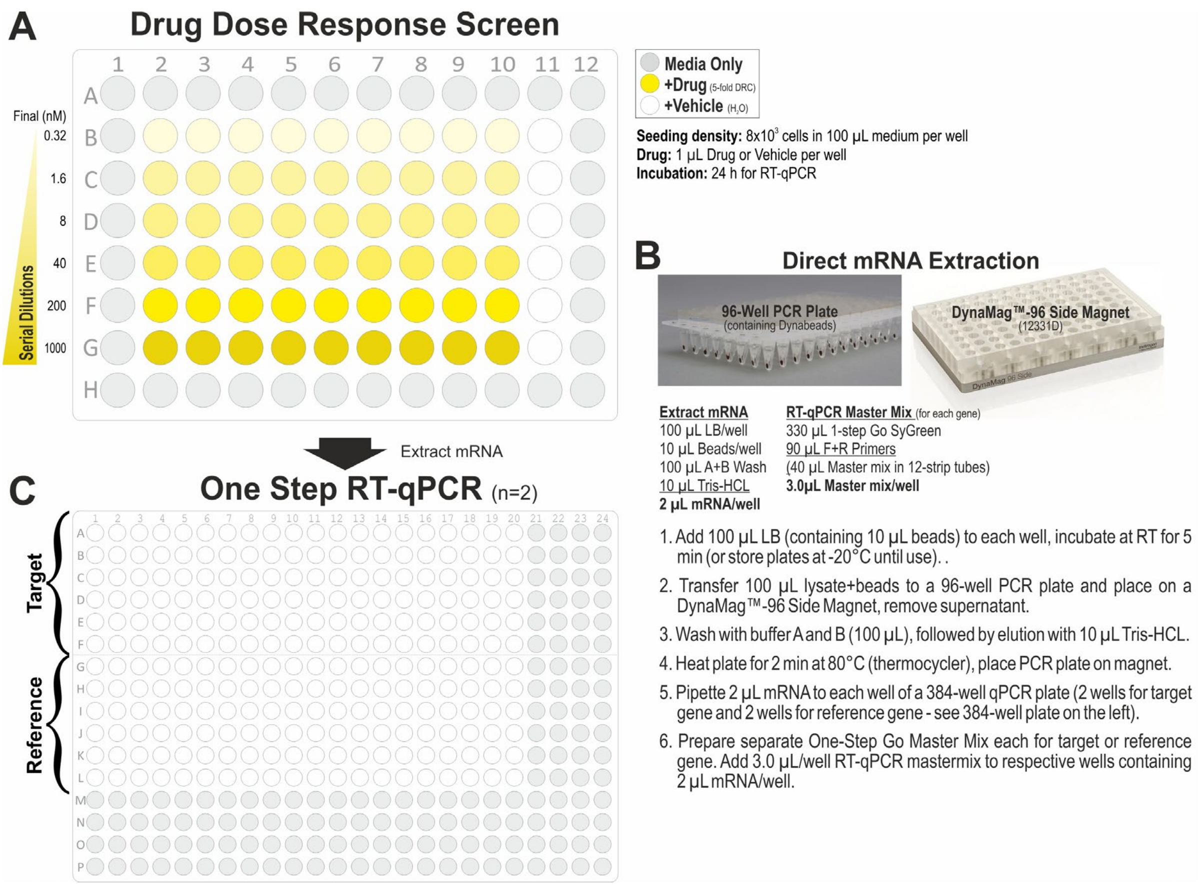

Figure S4. *Drug-gene dose-dependent interaction screening protocol and plate setups. A. Cells (8x10^3 cells/well) were treated for 24h with serial dilution series of nine different drugs as indicated in the diagram. Wells along the edges were not used to eliminate non-specific edge-associated effects. Control cells were treated with equal volume of vehicle (distilled water). B. Direct mRNA extraction method using Dynabeads were performed using a 96-well PCR plate and DynaMag™-96 Side Magnet (12331D) with protocol as illustrated. A total of 60 mRNA samples harvested from panel A (using protocol in panel B) were simultaneously extracted for RT-qPCR in panel C. C. One-step RT-qPCR 384-well plate map for target and reference gene expression quantification in the 60 mRNA samples performed in duplicates.*

Statistical Analysis

Statistical t-tests P values were used for differential analysis between two groups of data. Linear and non-linear regression analyses were used to quantify the relationship between serial drug concentrations and gene expression levels (Additional Data Figure S5-S16). Beeswarm Boxplots were created in R (version 2.13.1; The R Foundation for Statistical Computing) [20].

References

1. Teh MT, Hutchison IL, Costea DE, Neppelberg E, Liavaag PG, Purdie K, Harwood C, Wan H, Odell EW, Hackshaw A, Waseem A: **Exploiting FOXM1-orchestrated molecular network for early squamous cell carcinoma diagnosis and prognosis.** *Int J Cancer* 2013, **132:**2095-2106.
2. Ma H, Dai H, Duan X, Tang Z, Liu R, Sun K, Zhou K, Chen H, Xiang H, Wang J, et al: **Independent evaluation of a FOXM1-based quantitative malignancy diagnostic system (qMIDS) on head and neck squamous cell carcinomas.** *Oncotarget* 2016, **7:**54555-54563.
3. Teh MT, Ma H, Liang YY, Solomon MC, Chaurasia A, Patil R, Tekade SA, Mishra D, Qadir F, Yeung JS, et al: **Molecular Signatures of Tumour and Its Microenvironment for Precise Quantitative Diagnosis of Oral Squamous Cell Carcinoma: An International Multi-Cohort Diagnostic Validation Study.** *Cancers (Basel)* 2022, **14**.
4. Gemenetzidis E, Bose A, Riaz AM, Chaplin T, Young BD, Ali M, Sugden D, Thurlow JK, Cheong SC, Teo SH, et al: **FOXM1 upregulation is an early event in human squamous cell carcinoma and it is enhanced by nicotine during malignant transformation.** *PLoS One* 2009, **4:**e4849.
5. Teh MT, Gemenetzidis E, Chaplin T, Young BD, Philpott MP: **Upregulation of FOXM1 induces genomic instability in human epidermal keratinocytes.** *Mol Cancer* 2010, **9:**45.
6. Kulkarni PS, Sundqvist K, Betsholtz C, Hoglund P, Wiman KG, Zhivotovsky B, Bertolero F, Liu Y, Grafstrom RC: **Characterization of human buccal epithelial cells transfected with the simian virus 40 T-antigen gene.** *Carcinogenesis* 1995, **16:**2515-2521.
7. Rheinwald JG, Beckett MA: **Tumorigenic keratinocyte lines requiring anchorage and fibroblast support cultured from human squamous cell carcinomas.** *Cancer Res* 1981, **41:**1657-1663.
8. Reiss M, Pitman SW, Sartorelli AC: **Modulation of the terminal differentiation of human squamous carcinoma cells in vitro by all-trans-retinoic acid.** *J Natl Cancer Inst* 1985, **74:**1015-1023.
9. Locke M, Heywood M, Fawell S, Mackenzie IC: **Retention of intrinsic stem cell hierarchies in carcinoma-derived cell lines.** *Cancer Res* 2005, **65:**8944-8950.
10. Gemenetzidis E, Elena-Costea D, Parkinson EK, Waseem A, Wan H, Teh MT: **Induction of human epithelial stem/progenitor expansion by FOXM1.** *Cancer Res* 2010, **70:**9515-9526.
11. Qadir F, Aziz MA, Sari CP, Ma H, Dai H, Wang X, Raithatha D, Da Silva LGL, Hussain M, Poorkasreiy SP, et al: **Transcriptome reprogramming by cancer exosomes: identification of novel molecular targets in matrix and immune modulation.** *Mol Cancer* 2018, **17:**97.
12. Bustin SA, Benes V, Garson JA, Hellemans J, Huggett J, Kubista M, Mueller R, Nolan T, Pfaffl MW, Shipley GL, et al: **The MIQE guidelines: minimum information for publication of quantitative real-time PCR experiments.** *Clin Chem* 2009, **55:**611-622.
13. Zhao S, Fernald RD: **Comprehensive algorithm for quantitative real-time polymerase chain reaction.** *J Comput Biol* 2005, **12:**1047-1064.
14. Vandesompele J, De Preter K, Pattyn F, Poppe B, Van Roy N, De Paepe A, Speleman F: **Accurate normalization of real-time quantitative RT-PCR data by geometric averaging of multiple internal control genes.** *Genome Biol* 2002, **3:**RESEARCH0034.
15. DeLean A, Munson PJ, Rodbard D: **Simultaneous analysis of families of sigmoidal curves: application to bioassay, radioligand assay, and physiological dose-response curves.** *Am J Physiol* 1978, **235:**E97-102.
16. Rhodes DR, Yu J, Shanker K, Deshpande N, Varambally R, Ghosh D, Barrette T, Pandey A, Chinnaiyan AM: **ONCOMINE: a cancer microarray database and integrated data-mining platform.** *Neoplasia* 2004, **6:**1-6.
17. Nagy A, Lanczky A, Menyhart O, Gyorffy B: **Validation of miRNA prognostic power in hepatocellular carcinoma using expression data of independent datasets.** *Sci Rep* 2018, **8:**9227.
18. Tang Z, Li C, Kang B, Gao G, Li C, Zhang Z: **GEPIA: a web server for cancer and normal gene expression profiling and interactive analyses.** *Nucleic Acids Res* 2017, **45:**W98-W102.
19. Consortium GT: **Human genomics. The Genotype-Tissue Expression (GTEx) pilot analysis: multitissue gene regulation in humans.** *Science* 2015, **348:**648-660.
20. Juul N, Szallasi Z, Eklund AC, Li Q, Burrell RA, Gerlinger M, Valero V, Andreopoulou E, Esteva FJ, Symmans WF, et al: **Assessment of an RNA interference screen-derived mitotic and ceramide pathway metagene as a predictor of response to neoadjuvant paclitaxel for primary triple-negative breast cancer: a retrospective analysis of five clinical trials.** *Lancet Oncol* 2010, **11:**358-365.
