# Supplementary material for: Identification of multidrug chemoresistant genes in head and neck squamous cell carcinoma cells
Source: Mol Cancer. 2023 Sep 4;22:146. doi: 10.1186/s12943-023-01846-3 (PMC10476423; doi:10.1186/s12943-023-01846-3)
Supplement: Supplementary file 2 — Supplementary Material 2 [file 12943_2023_1846_MOESM2_ESM.pdf]

Supplementary Information

Table S1 – Summary of HNSCC Microarray Datasets

Anatomical Sites	PMID[a]	GEO[b]	Tumour	Normal	LNM[c]
Nasopharynx	16912175	GSE12452	31	10	
Hypopharynx	14676830	GSE2379	34	4	
Hypopharynx	16205657	GSE1722	6	4	2
Tongue	19138406	GSE13601	37	20	
Tongue	15170515	GSE6631	22	22	
Tongue	18254958	GSE9844	53	22	
Oral Cavity	15381369	GSE3524	16	4	
Oral Cavity & Larynx	14729608	Ginos HN*	41	13	

[a]PubMed ID; [b]Gene Expression Omnibus accession number; [c]Lymph Node Metastasis; *Oncomine Dataset name.

Table S2 - Biomarker Primer Sequences

Gene	Loci	Forward Primer	Reverse Primer	Bp[a]
**BIRC5**	17q25.3	AGAACTGGCCCTTCTTGGA	ACACTGGGCCAAGTCTGG	104
**BUB1B**	15q15.1	CAGTCAGACTCTCAGCATCAAGA	CGAGGCAGAAGAACCAGAGA	94
**CBX7**	22q13.1	CGAGTATCTGGTGAAGTGGAAA	GGGGGTCCAAGATGTGCT	77
**CDCA5**	11q13.1	AGACATGACTCTCCCTGGAATC	CACTCATCCAGCTCCGTTTT	96
**CENPA**	2p23.3	CTGCACCCAGTGTTTCTGTC	GAGAGTCCCCGGTATCATCC	63
**CLEC3B**	3p21.31	AGCAGCATGGAGCTCTGG	CTCCTCAAACATCTTTGTGTTCA	144
**CRNN**	1q21.3	GAGCAAGAGTTTGCCGATGT	TCCACAGTCCCTGTGTGGT	98
**CXCL8**	4q13.3	AAGTTTTTGAAGAGGGCTGAGA	TGGCATCTTCACTGATTCTTGGA	74
**DNMT1**	19p13.2	CGATGTGGCGTCTGTGAG	TGTCCTTGCAGGCTTTACATT	64
**DUOX1**	15q21.1	GGAGGTTTGGCAAGAAGGT	GCGCTTGAACTGTTGCAC	110
**FN1**	2q35	AACGTGGGAGAAGCCCTAC	TTGTGTCCTGATCGTTGCAT	113
**FOXM1**	12p13.33	ACTTTAAGCACATTGCCAAGC	CGTGCAGGGAAAGGTTGT	63
**FOXM1B**	12p13.33	CCAGGTGTTTAAGCAGCAGA	TCCTCAGCTAGCAGCACCTTG	279
**FOXO1**	13q14.11	AGGCTGAGGGTTAGTGAGCA	TGAAAGACATCTTTGGACTGCTT	91
**FOXO3**	6q21	TTCAAGGATAAGGGCGACAG	CGACTATGCAGTGACAGGTTG	77
**FOXO4**	Xq13.1	ACGAGTGGATGGTCCGTACT	GTGGCGGATCGAGTTCTTC	86
**FOXO6**	1p34.2	AAGGATAAAGGCGACAGCAA	GTGTGCAGCGACAGGTTG	71
**HOXA7**	7p15.2	GCCAATTTCCGCATCTACCC	GGTAGCGGTTGAAGTGGAAC	121
**INHBA**	7p14.1	GCTCAGACAGCTCTTACCACA	AAATTCTCTTTCTGGTCCCCACT	69
**IVL**	1q21.3	TGCCTGAGCAAGAATGTGAG	TTCCTCATGCTGTTCCCAGT	83
**MMP13**	11q22.2	TGAGCTGGACTCATTGTCGG	AGGTAGCGCTCTGCAAACTG	94
**NEK2**	1q32.3	CATTGGCACAGGCTCCTAC	GAGCCATAGTCAAGTTCTTTCCA	90
**NR3C1**	5q31.3	TCCCTGGTCGAACAGTTTTT	GCTGGATGGAGGAGAGCTTA	77
**PLAU**	10q22.2	TCACTGGCTTTGGAAAAGAGA	TGGTGACTTCAGAGCCGTAG	126
**S100A16**	1q21.3	CAAGATCAGCAAGAGCAGCTT	GAGCTTATCCGCAGCCTTC	94
**SIRT1**	10q21.3	AAATGCTGGCCTAATAGAGTGG	TGGCAAAAACAGATACTGATTACC	75
**TOP2A**	17q21.2	CAGTGAAGAAGACAGCAGCAAA	AAGCTGGATCCCTTTTAGTTCC	96
**VIM**	10p13	AGGTGGACCAGCTAACCAAC	TTTCGGCTTCCTCTCTCTGA	123
**POLR2A***	17p13.1	TCCGTATTCGCATCATGAAC	TCATCCATCTTGTCCACCAC	73
**YAP1***	11q22.1	ACAATGACGACCAATAGCTCAG	CCACTGTCTGTACTCTCATCTCG	77

*Reference genes

Figure S1

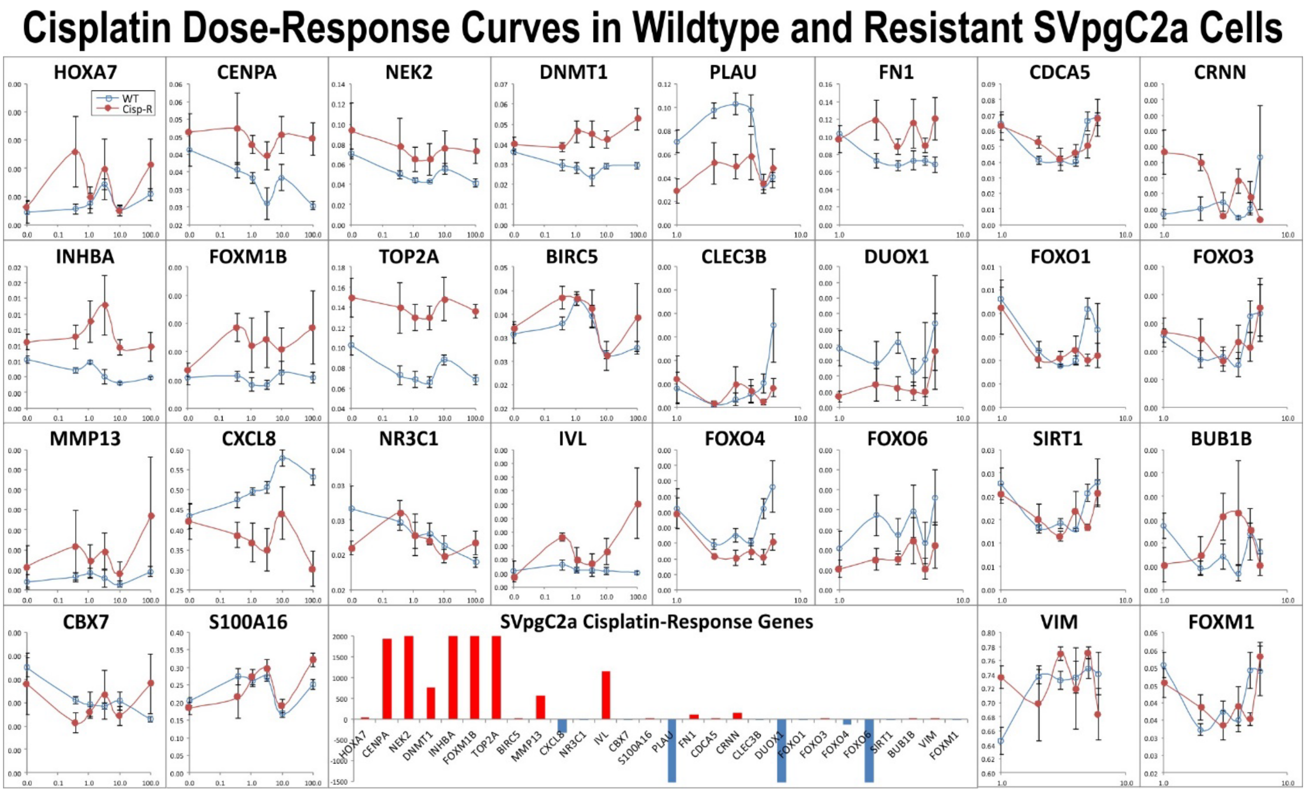

Figure S2

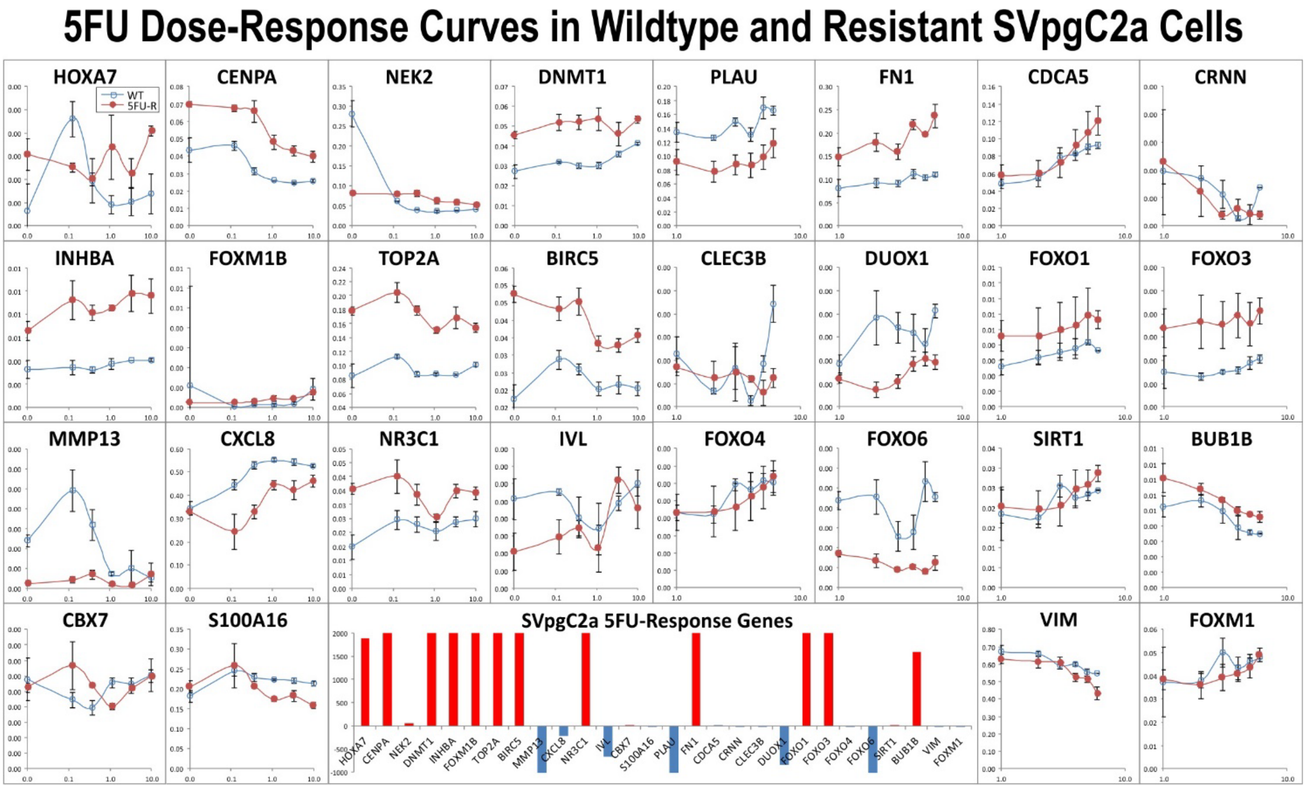

Figure S3

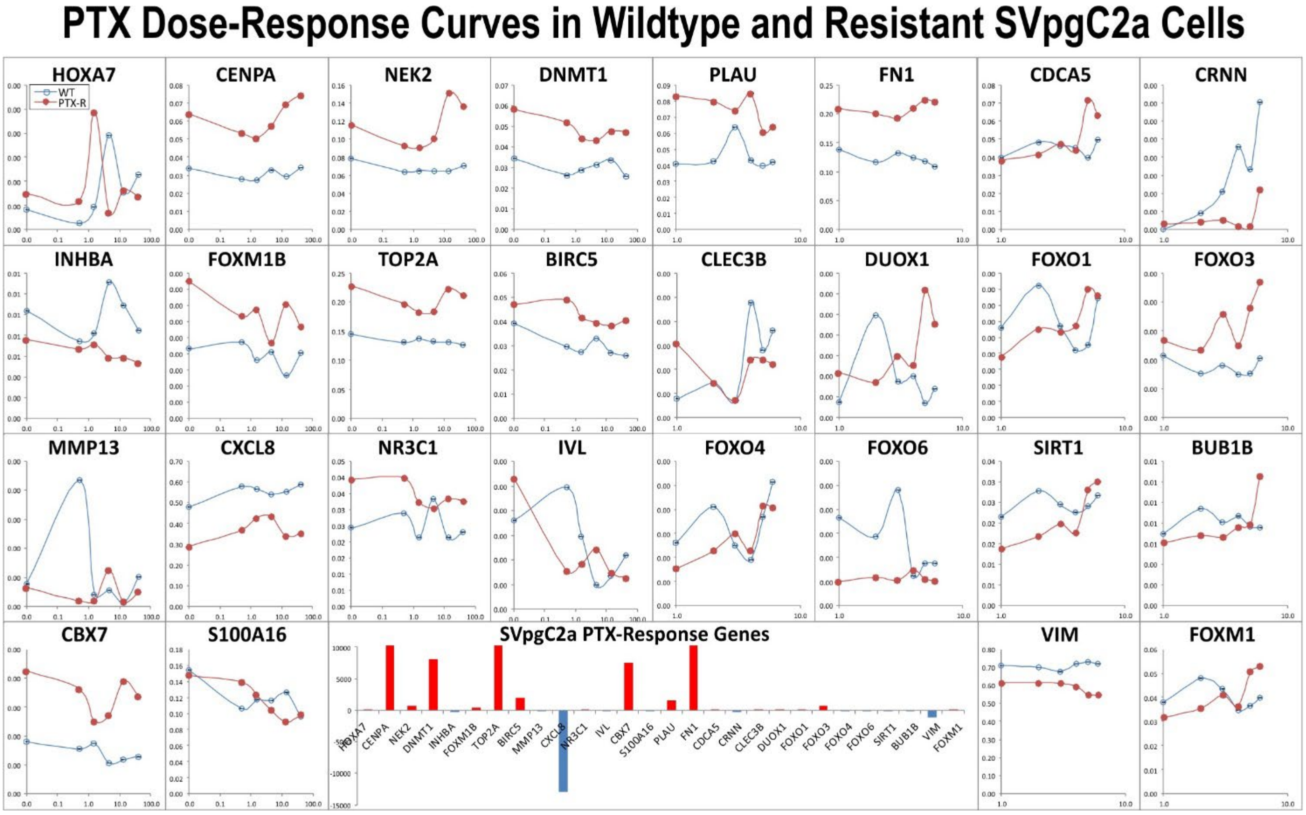

Figure S4

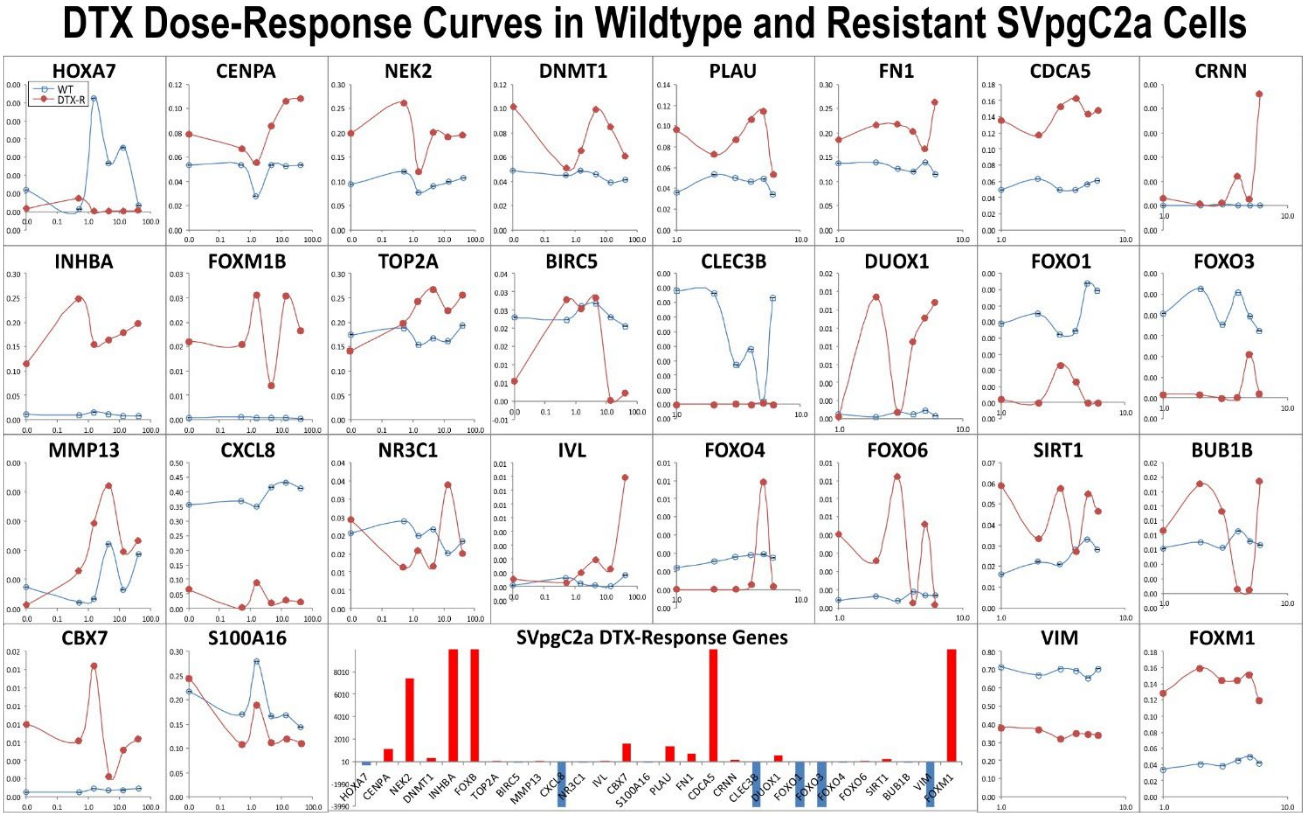

Figure S5

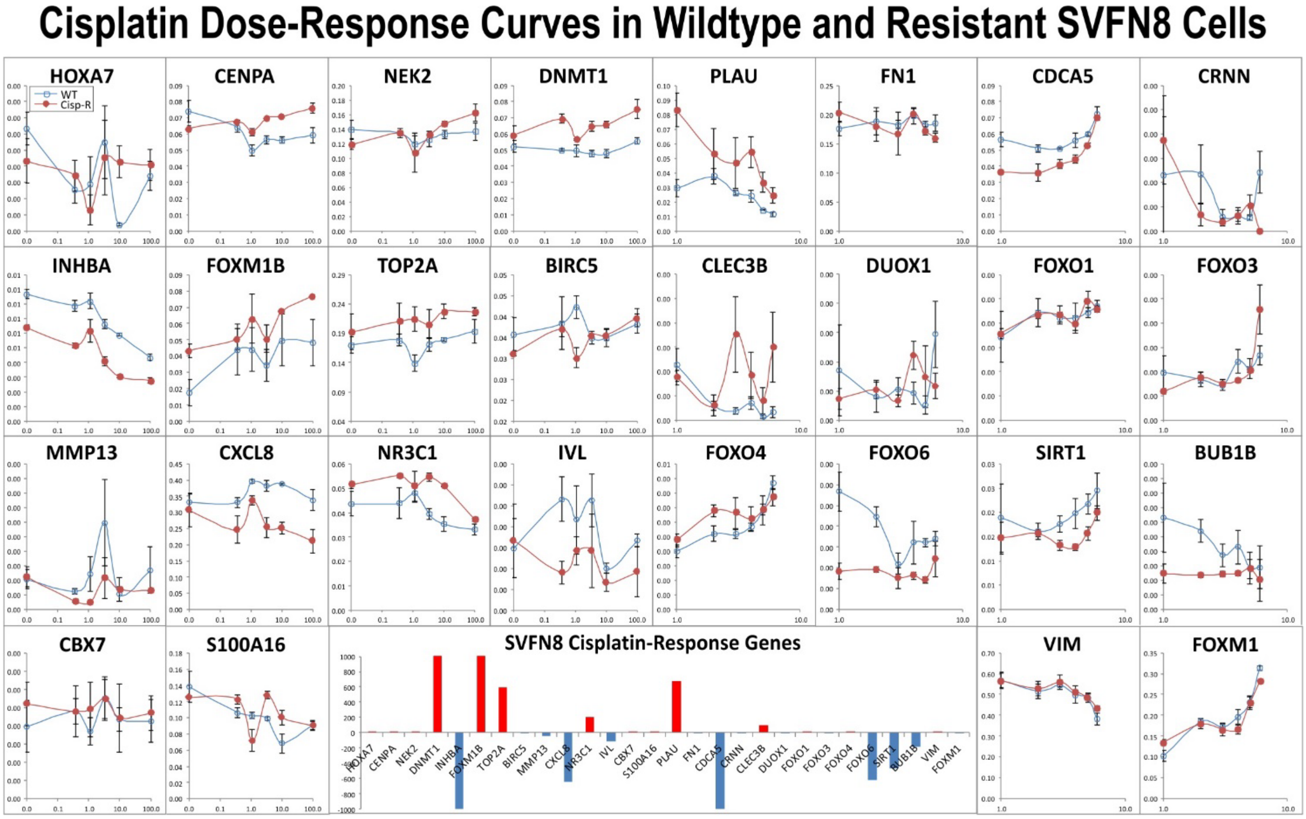

Figure S6

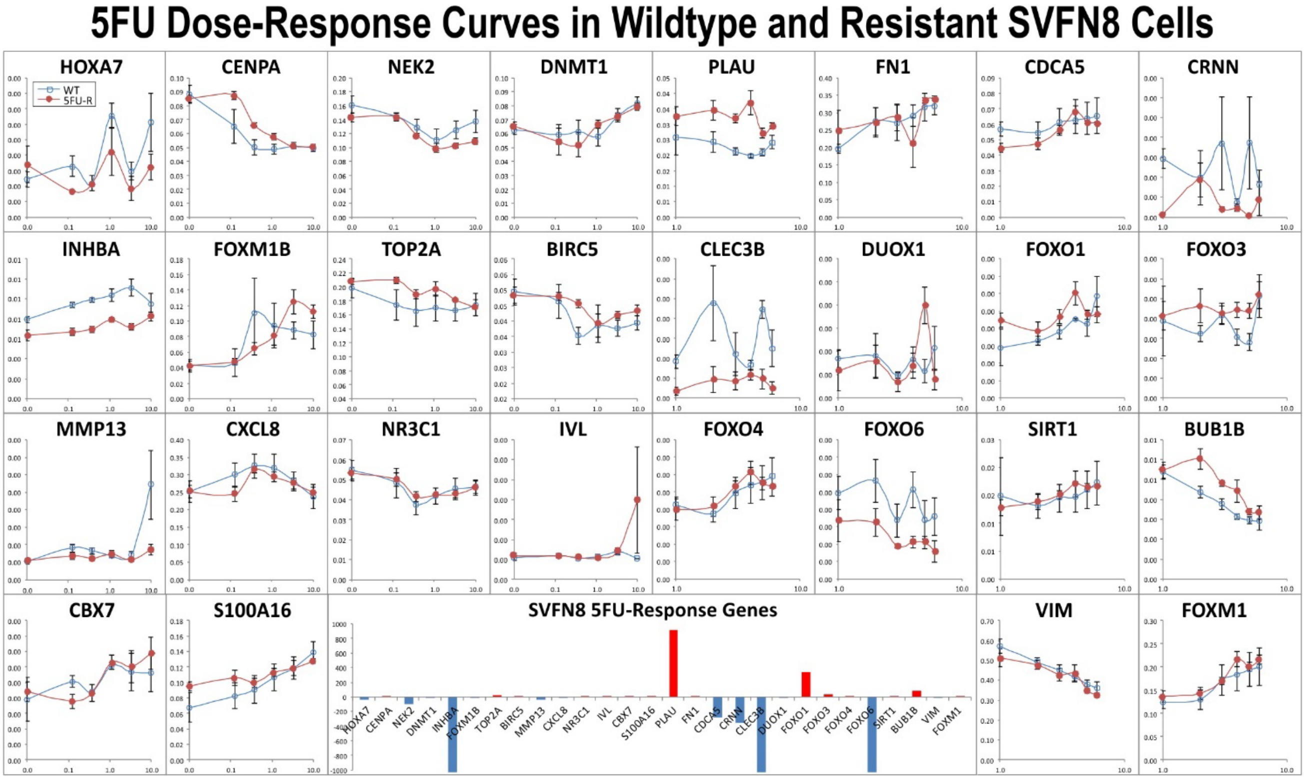

Figure S7

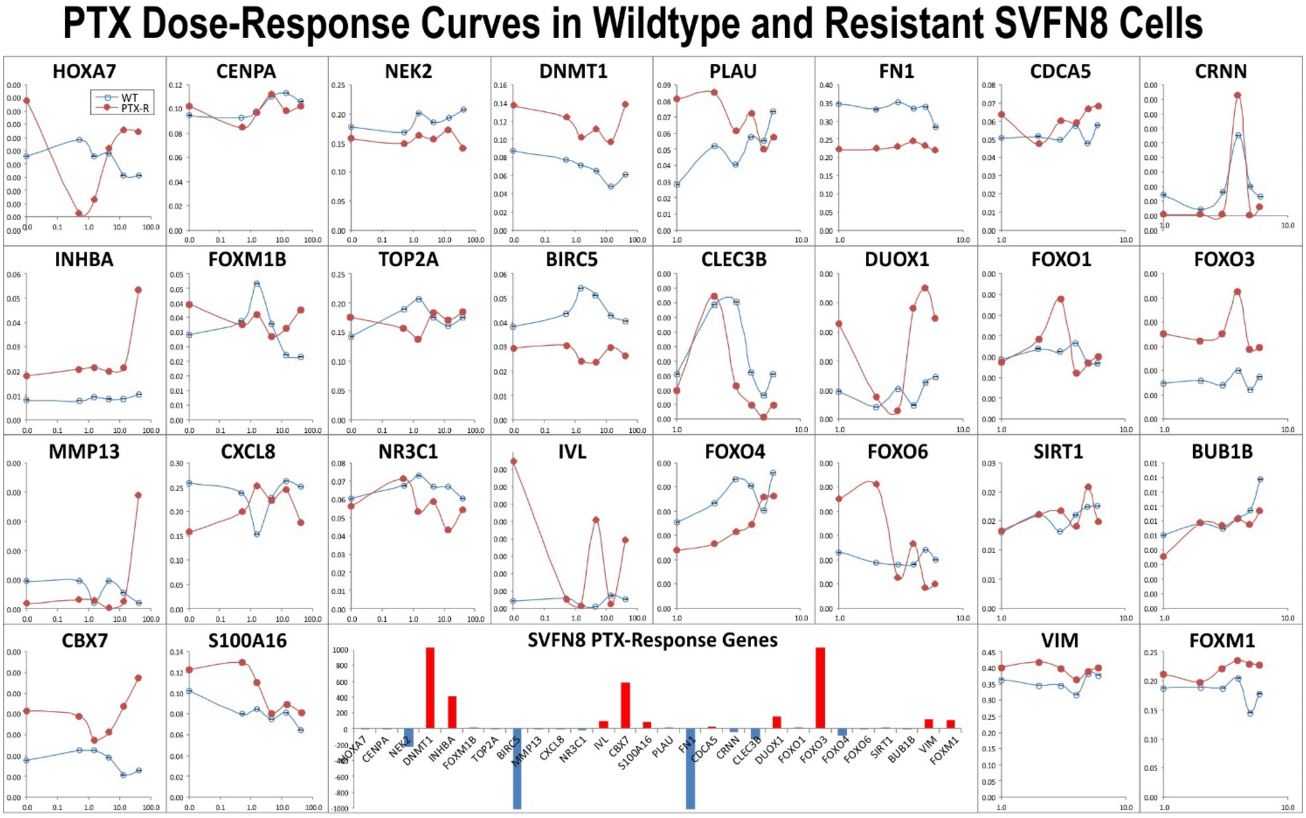

Figure S8

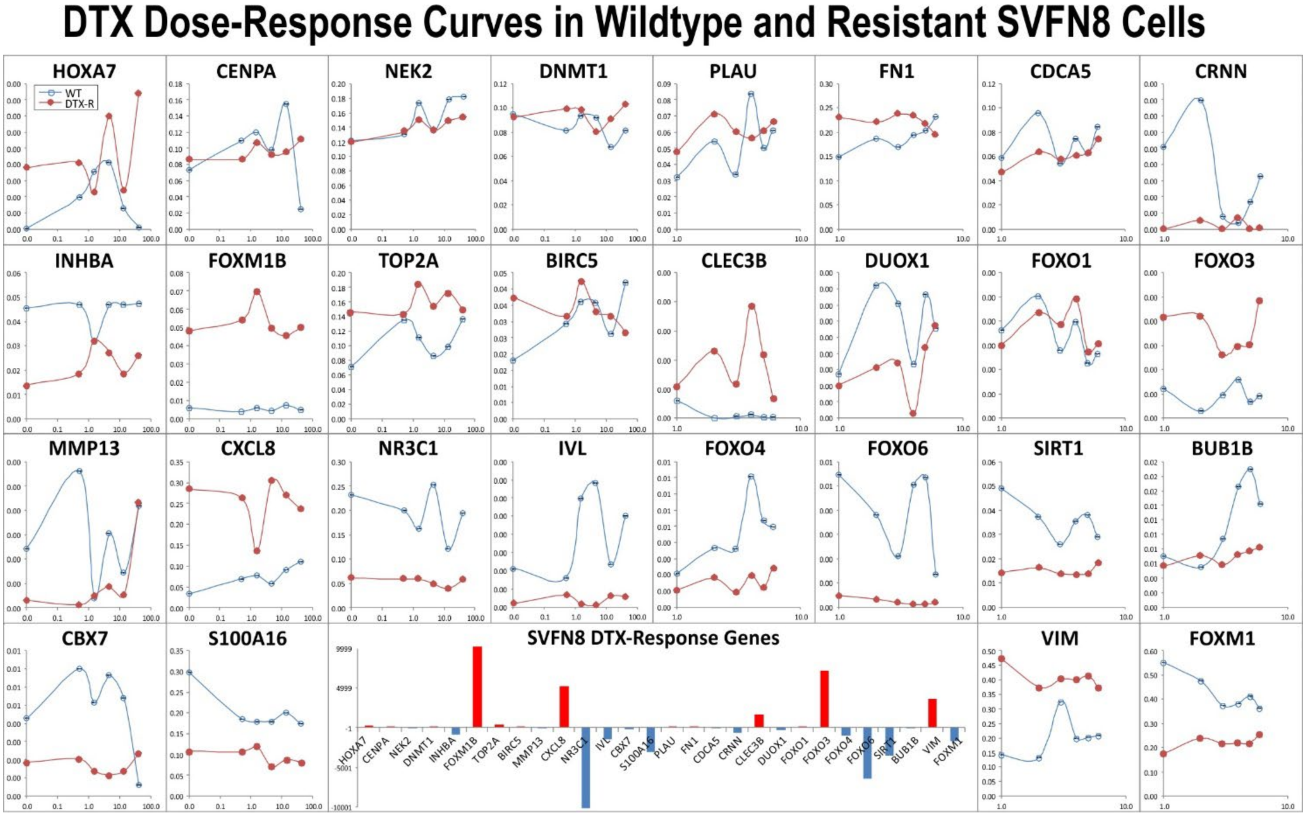

Figure S9

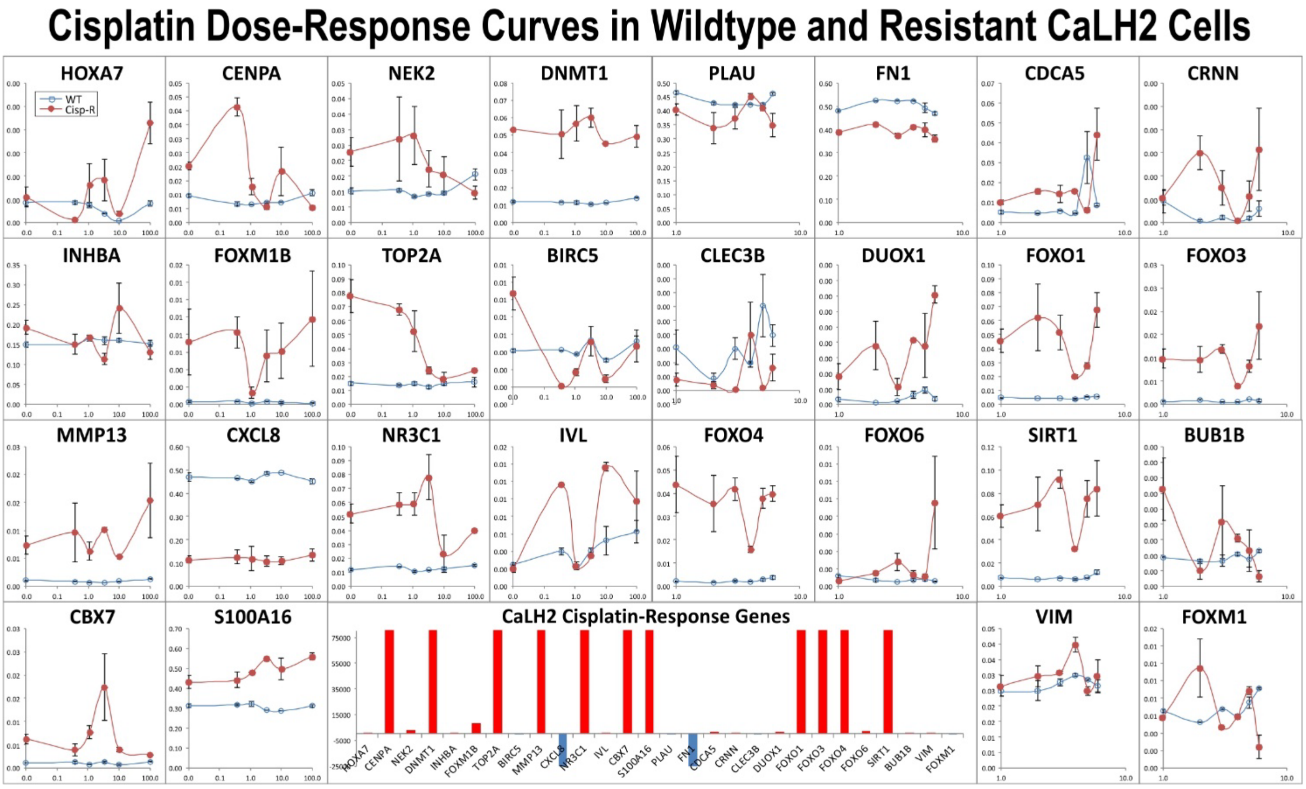

Figure S10

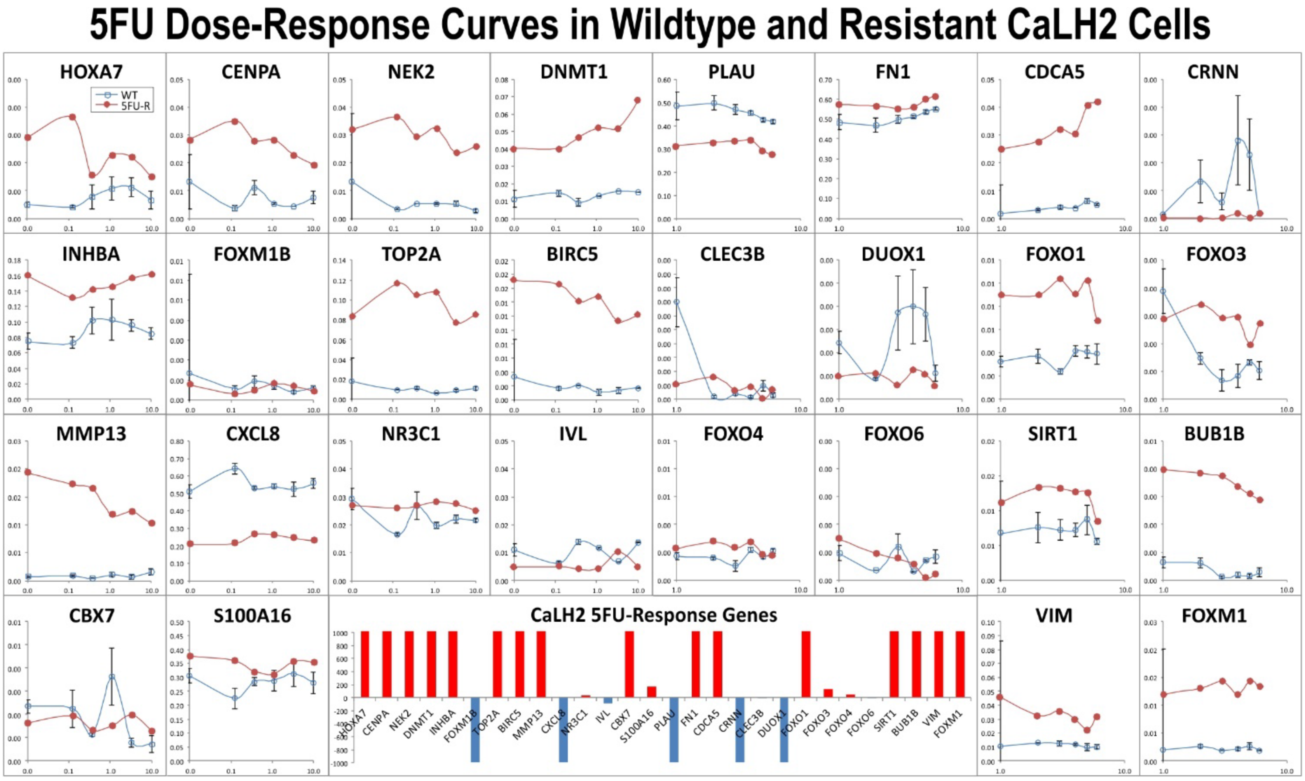

Figure S11

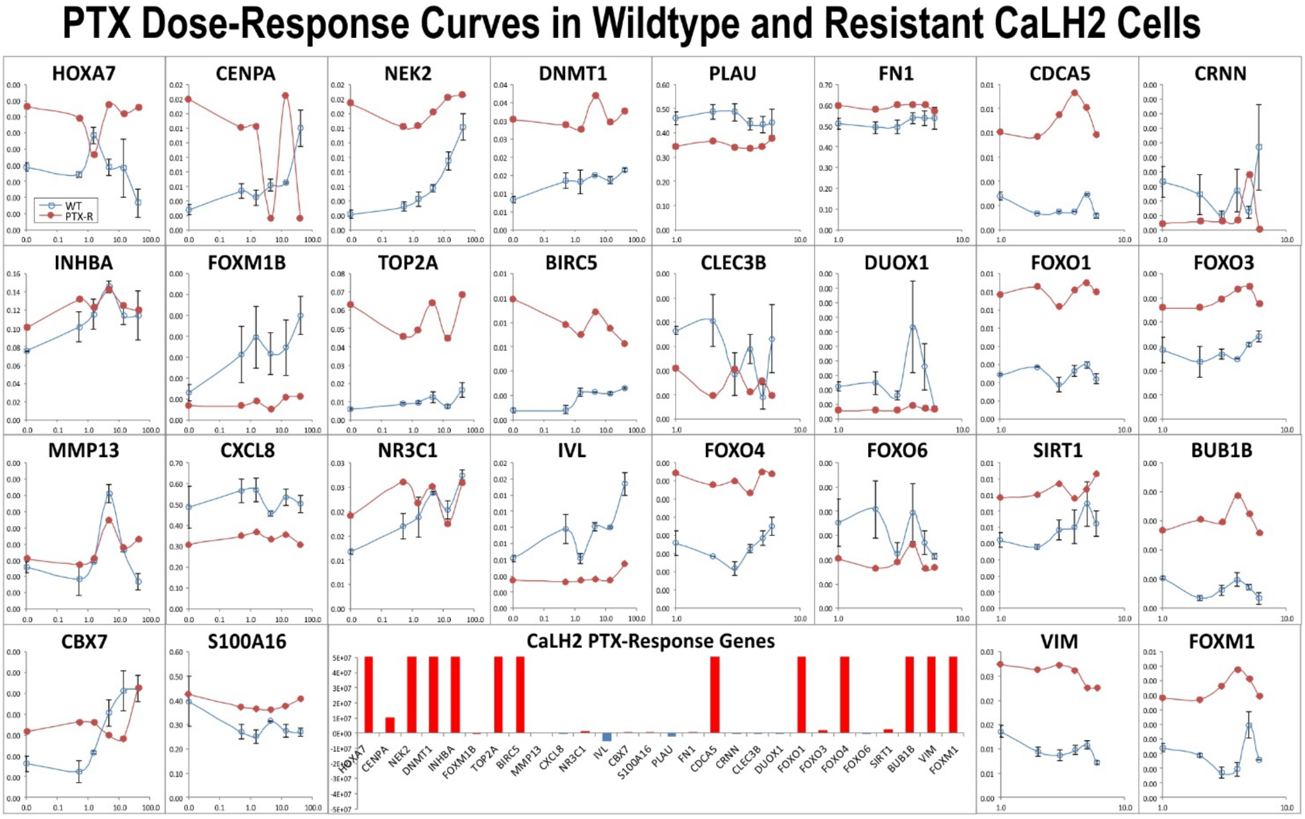

Figure S12

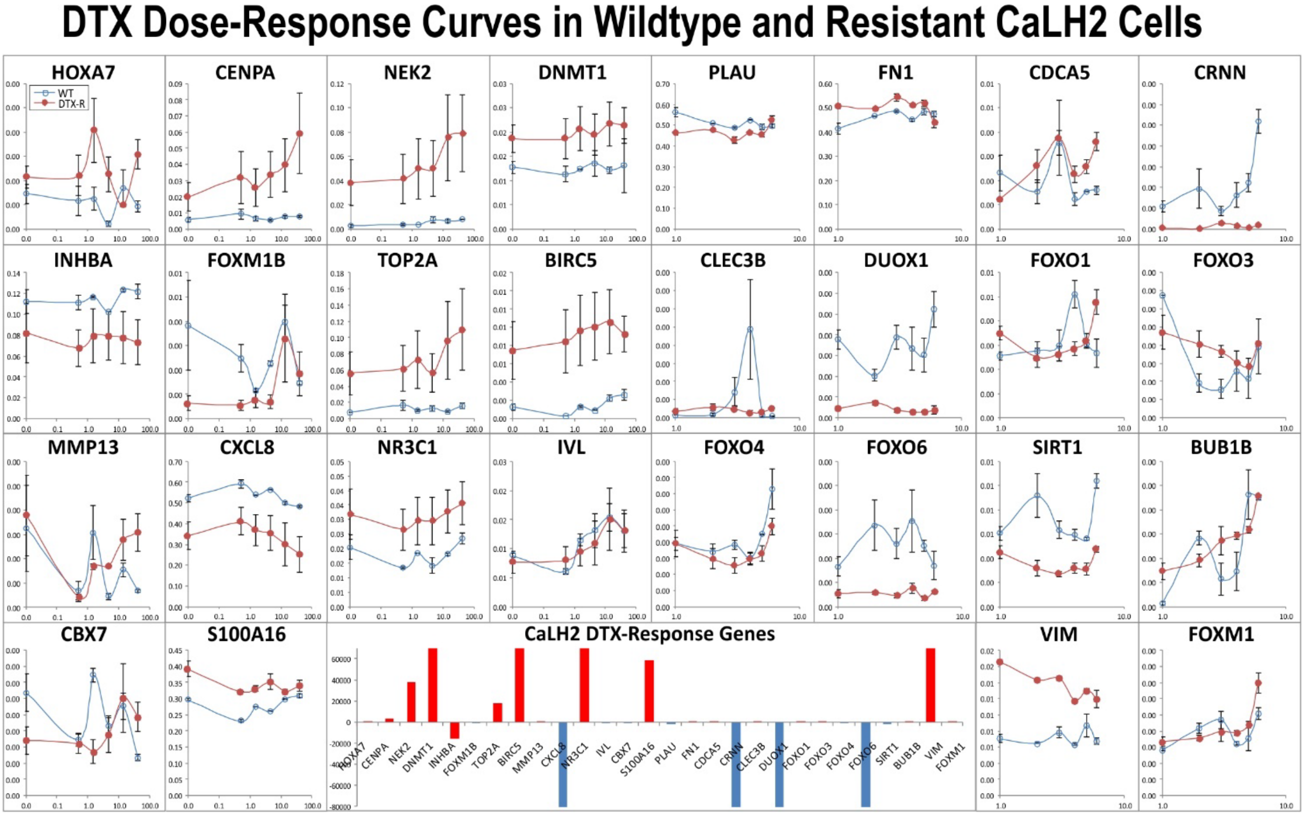

Figure S1-S12. *Differential gene expression analysis on 12 chemoresistant cell strains vs wildtype cells in response to corresponding drugs (cisplatin, 5FU, PTX and DTX). Wildtype (WT; blue line) and drug-resistant (R; red line) cells were treated with serial dilution of corresponding drug for 24h prior to harvest for RT-qPCR to quantify mRNA expression levels of the 28 genes. Assay format and drug concentrations are shown in Additional File 1. Relative gene expression (Y-axis) were plotted against logarithmic drug concentrations (X-axis). Each data point represents a mean of quadruplicates with corresponding SEM error bars. For each gene, the total area between WT and R curves were calculated based on t-test P-values and regression analyses. The most significant up (red bars) or down-regulated (blue bars) genes between WT and R cells are shown as a bar chart in the middle-bottom panel. The significant genes are ranked and tabulated in Figure 1B.*

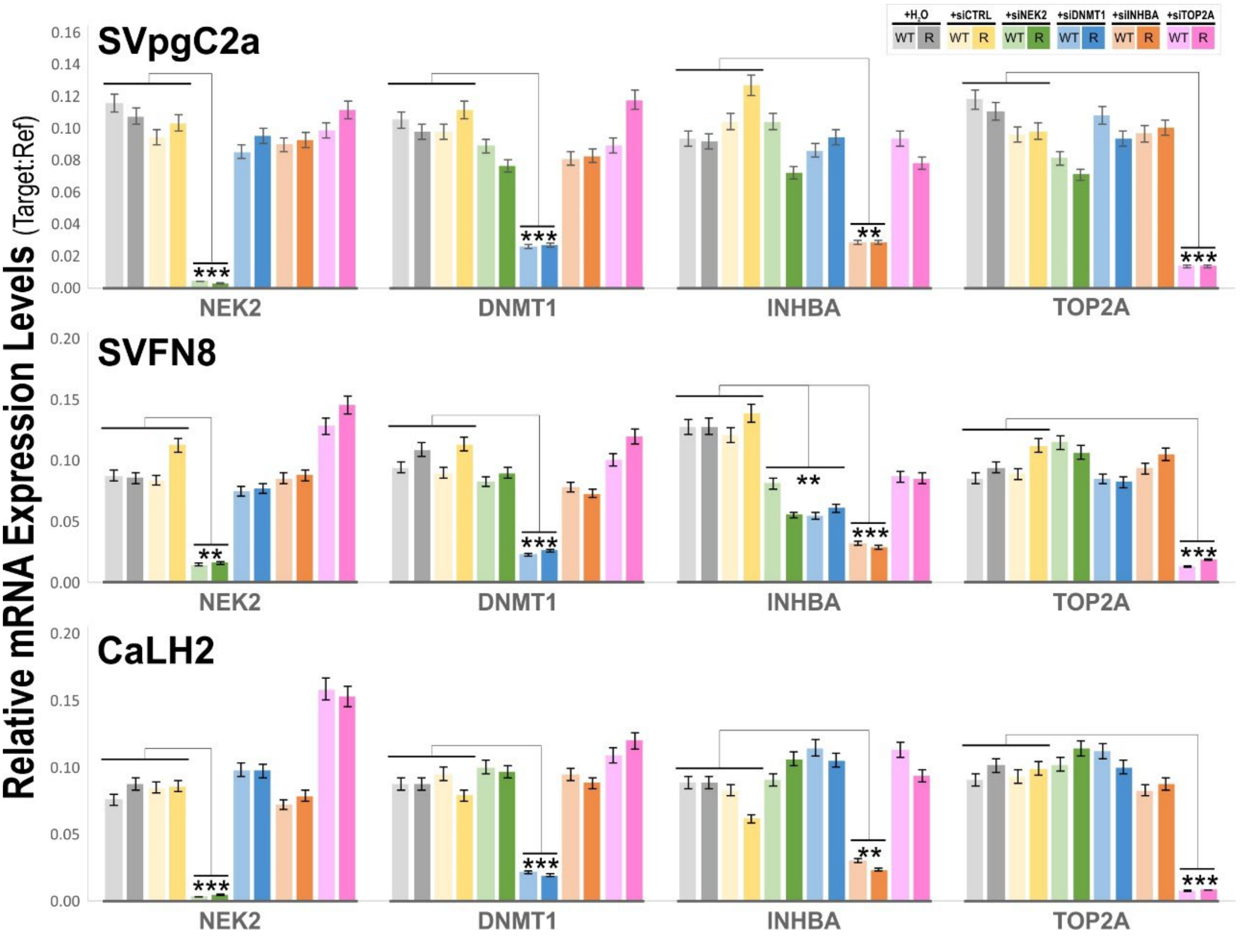

Figure S13. *Validation of gene-specific mRNA knockdown by corresponding siRNA in all 12 cell strains using RT-qPCR. Wildtype (WT) and drug-resistant cells (R) were transfected by either H2O (untransfected control), siCTRL, siNEK2, siDNMT1, siINHBA or siTOP2A as indicated for 3 days followed by RT-qPCR to measure the relative mRNA expression levels of NEK2, DNMT1, INHBA and TOP2A. All 4 siRNAs showed statistically significant gene silencing on corresponding genes (**P<0.01; ***P<0.001) compared to both controls (H2O and siCTRL). None of the siRNAs showed any off-target effects apart from siINHBA on SVFN8 cell lines whereby, in addition to INHBA, NEK2 and DNMT1 mRNA levels were partially but significantly downregulated (**P<0.01). Assay protocol for siRNA transfection and drug concentrations are shown in Additional File 1.*

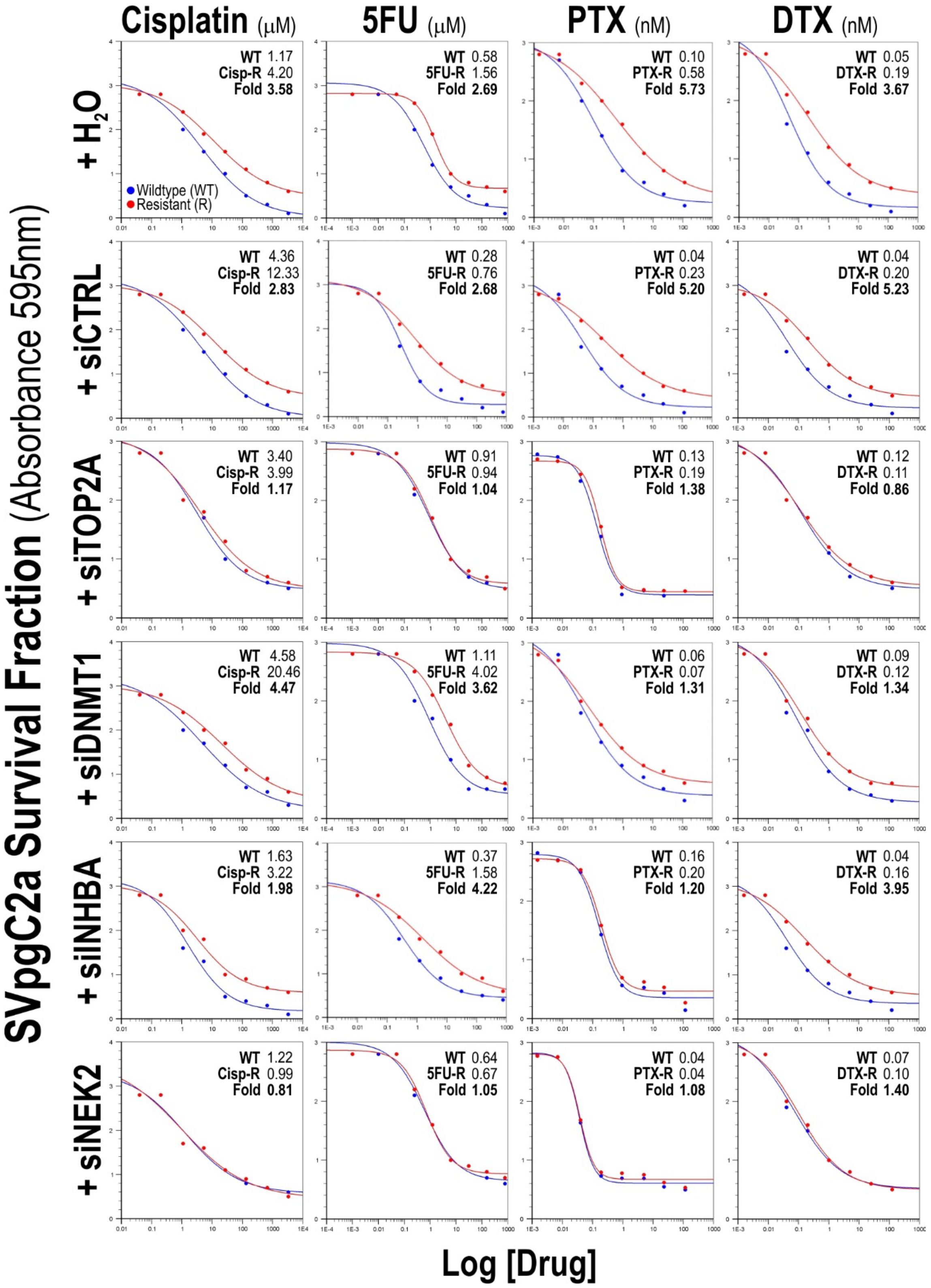

Figure S14. *Effects of siRNA gene silencing of TOP2A, DNMT1, INHBA and NEK2 on chemoresistance in SVpgC2a cells. IC_{50} values of each chemotherapeutic drugs on wildtype (WT) and chemoresistant (R) cells were determined using sigmoid-curve fitting algorithm on data points plotted as logarithmic drug concentrations on the X-axis and survival fraction (Absorbance at 595nm) on the Y-axis. IC_{50} fold differences were calculated between WT and R cells for each treatment as indicated within each graph panel. Assay protocol for siRNA transfection and drug concentrations are shown in Additional File 1.*

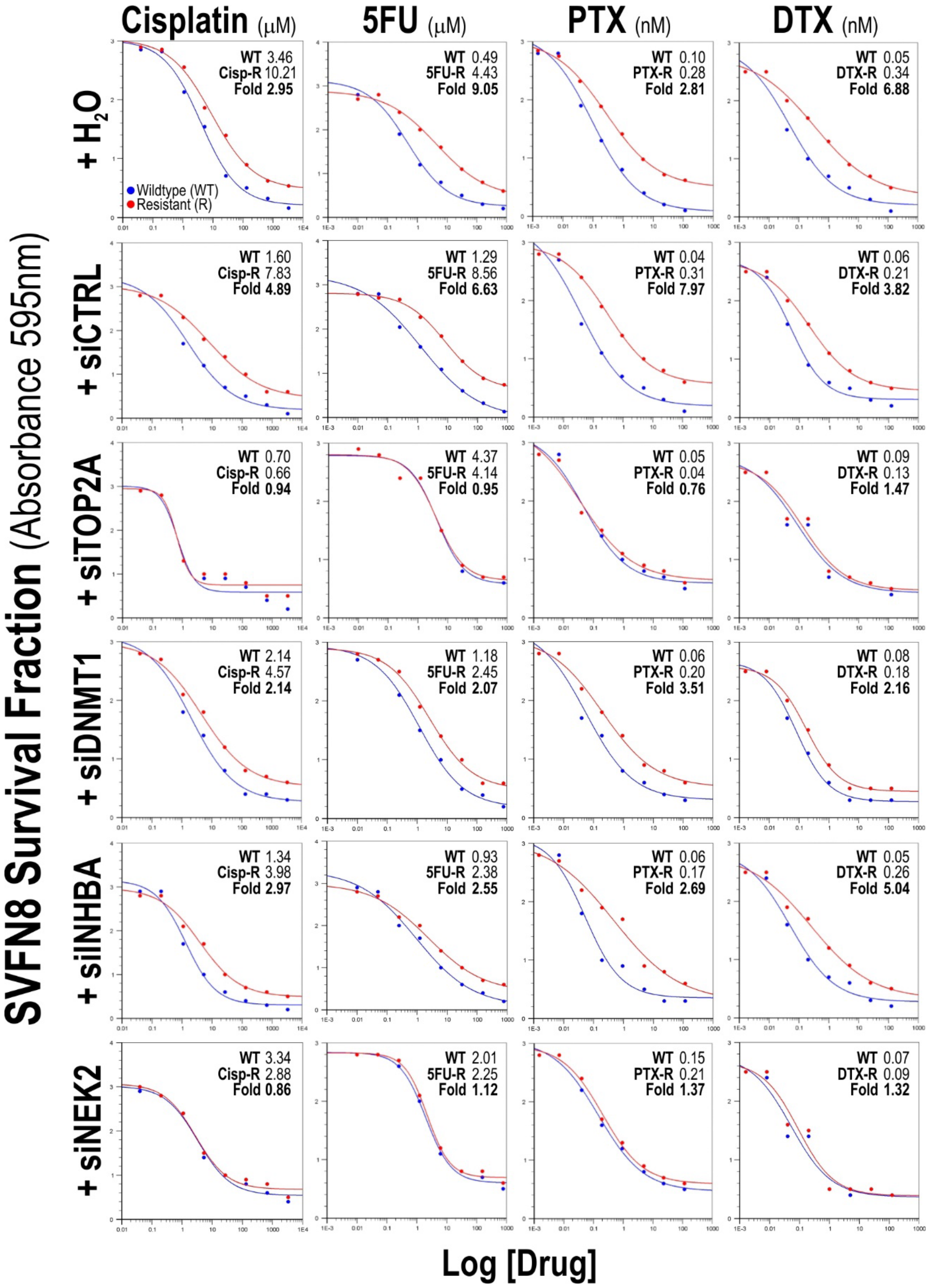

Figure S15. *Effects of siRNA gene silencing of TOP2A, DNMT1, INHBA and NEK2 on chemoresistance in SVFN8 cells. IC_{50} values of each chemotherapeutic drugs on wildtype (WT) and chemoresistant (R) cells were determined using sigmoid-curve fitting algorithm on data points plotted as logarithmic drug concentrations on the X-axis and survival fraction (Absorbance at 595nm) on the Y-axis. IC_{50} fold differences were calculated between WT and R cells for each treatment as indicated within each graph panel. Assay protocol for siRNA transfection and drug concentrations are shown in Additional File 1.*

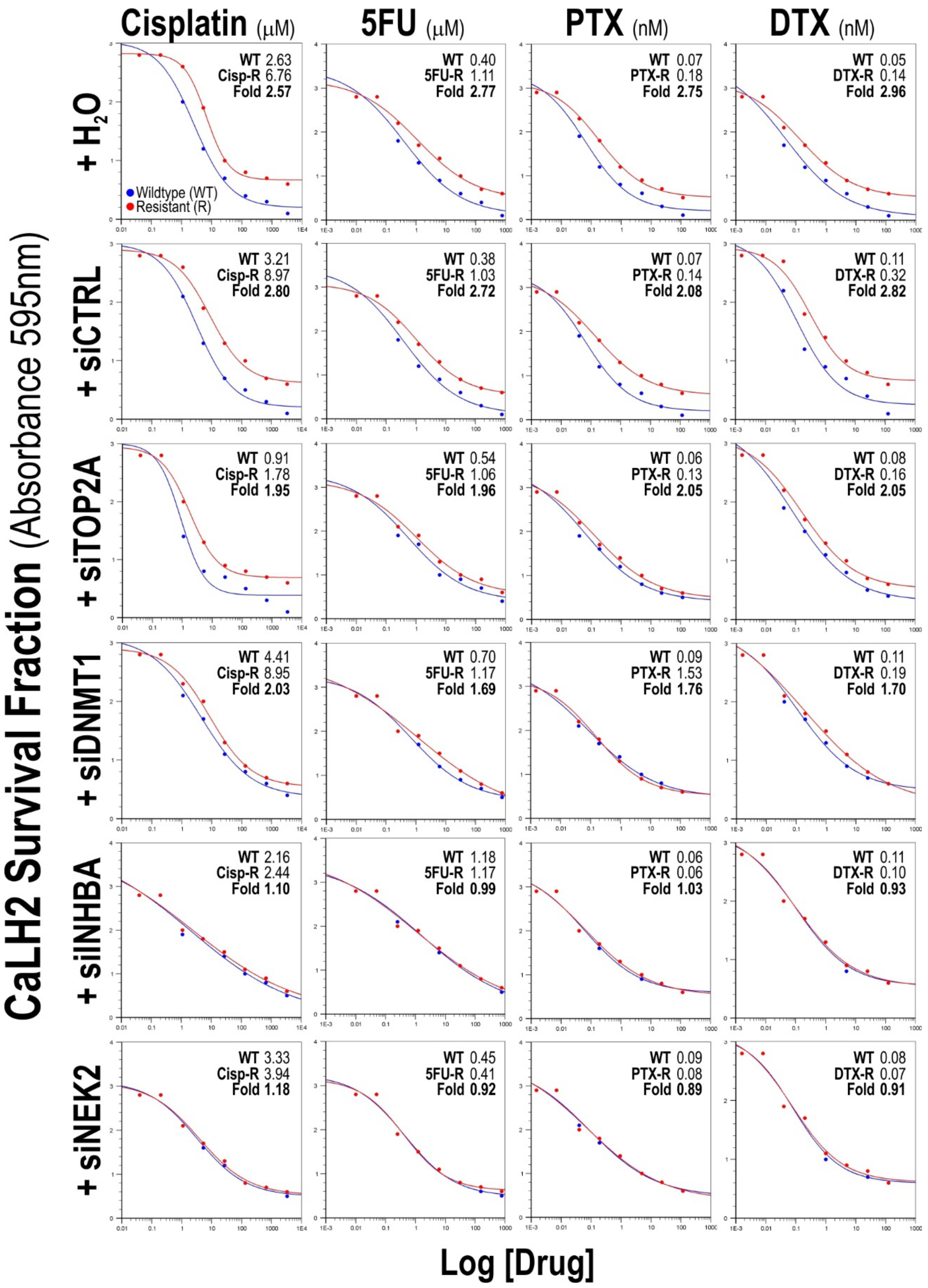

Figure S16. *Effects of siRNA gene silencing of TOP2A, DNMT1, INHBA and NEK2 on chemoresistance in CaLH2 cells. IC_{50} values of each chemotherapeutic drugs on wildtype (WT) and chemoresistant (R) cells were determined using sigmoid-curve fitting algorithm on data points plotted as logarithmic drug concentrations on the X-axis and cell viability (Absorbance at 595nm) on the Y-axis. IC_{50} fold differences were calculated between WT and R cells for each treatment as indicated within each graph panel. Assay protocol for siRNA transfection and drug concentrations are shown in Additional File 1.*

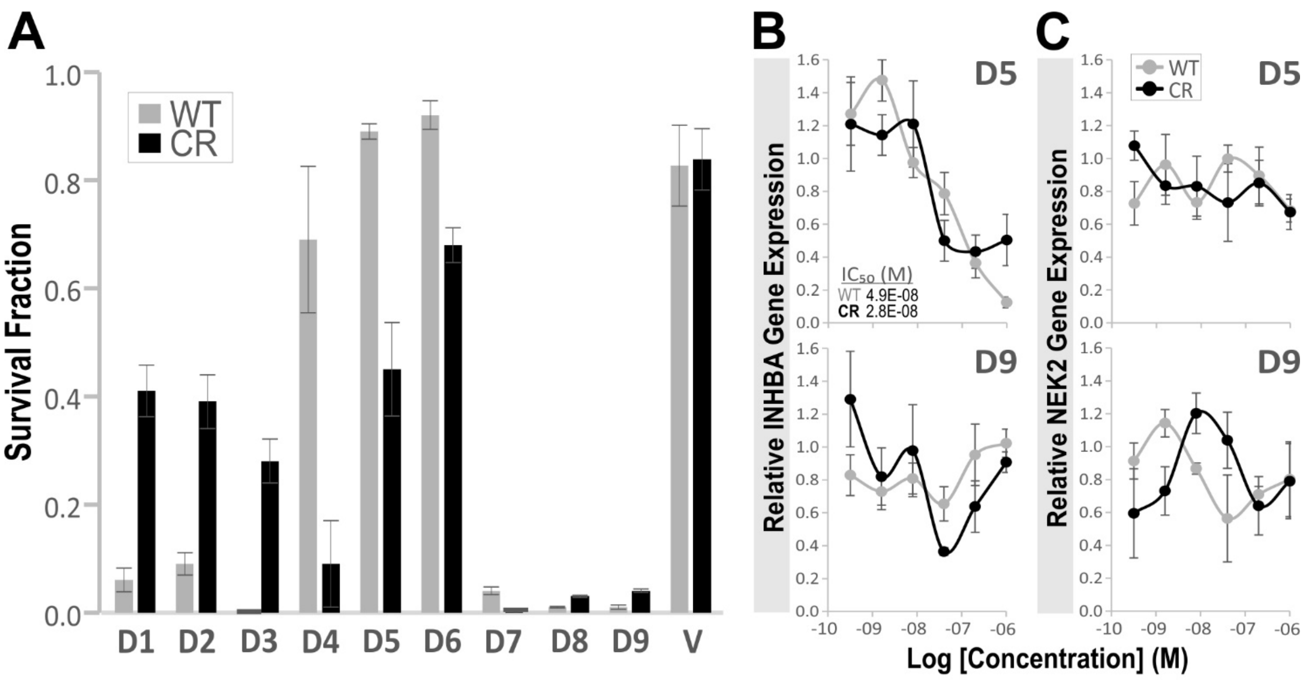

D

	NSC	Chemical name	CAS number	Mol. Formula	Mol. Weight
**D1**	407806	Digitoxigenin	CAS 143-62-4	C23H34O4	375
**D2**	761068	Cabozantinib	CAS 849217-68-1	C28H24FN3O5	502
**D3**	30625	Lasiocarpine	CAS 303-34-4	C21H33NO7	411
**D4**	294408	Sirodesmin A	CAS 52988-50-8	C20H26N2O8S2	487
**D5**	44138	1-O-phosphonohexopyranose	CAS 6799-01-5	C6H13O9P	260
**D6**	121849	CID5351990	N/A	C24H28N2O5	425
**D7**	758252	Carfilzomib	CAS 868540-17-4	C40H57N5O7	720
**D8**	269756	Baccharinol	CAS 63783-94-8	C29H38O11	563
**D9**	526417	Echinomycin A	CAS 512-64-1	C51H64N12O12S2	1101

Figure S17. *Drug library screen to identify drug-gene interactions for counteracting chemoresistance in HNSCC cells.* ***A****, A library of 537 compounds were screened using AlamarBlue cell viability assays on wildtype (WT) and cisplatin-resistant (CR) CaLH2 cell line performed in triplicates. Assay protocol details are shown in Additional File 1. Nine compounds (D1-D9) were selected based on the most significant growth inhibitory effects (t-test, p-value<0.05). Control cells were treated with vehicle (V; 1% DMSO).* ***B-C****, Dose-response assays for D5 and D9 on INHBA (****B****) and NEK2 (****C****) gene expression in WT and CR cells. Each datapoint represents relative gene expression (mean ± SEM) of quadruplicates quantified using RT-qPCR. Drug potencies (IC_{50}) on respective gene inhibition are displayed within each panel. Where dose-response curve fitting could not be performed, no IC50 value is shown.* ***D****, Basic chemical identities of D1-D9 drugs. NSC ID number is searchable at DTP Chemical database (https://dtp.cancer.gov/dtpstandard/ChemData/index.jsp).*
